# Mechanical Compatibility in Stitch Configuration and Sensor Adhesion for High‐Fidelity Pulse Wave Monitoring

**DOI:** 10.1002/advs.202415608

**Published:** 2025-02-14

**Authors:** Zhongda Chen, Jun Song, Yu Lu, Jing Zhu, Hongxu Zhu, Wenxian Du, Benhui Hu

**Affiliations:** ^1^ School of Biomedical Engineering and Informatics Nanjing Medical University 101 Longmian Avenue Nanjing 211166 China; ^2^ Materdicine Lab School of Life Sciences Shanghai University 99 Shangda Road Shanghai 200444 China; ^3^ School of Mechanical Engineering Nantong University 9 Seyuan Road Nantong 226002 China; ^4^ Department of Geriatrics Affiliated Nantong Hospital of Shanghai University (The Sixth People's Hospital of Nantong) Shanghai University 500 Yonghe Road Nantong 226011 China; ^5^ Institute of Diagnostic and Interventional Radiology Shanghai Sixth People's Hospital School of Medicine Shanghai Jiaotong University 600 Yishan Road Xuhui Shanghai 200233 China

**Keywords:** knitted structure, polyacrylamide hydrogel, pulse wave, strain sensor

## Abstract

Wearable electronics can achieve high‐fidelity monitoring of pulse waveforms on the body surface enabling early diagnosis of cardiovascular diseases (CVDs). Textile‐based wearable devices offer advantages in terms of high permeability and comfort. However, knitted strain sensors struggle to capture small‐range deformation signals due to stress dissipation during friction and slip of yarns within the textiles. They are optimized for mechanical adaptability and adhesive capability. In this work, the stitch configurations of knitted structure are adjusted to optimize the energy dissipation ratio during deformation and waveform fitting performance. These electric‐mechanical results enabled the selection of the most suitable knitted structure for the clinical diagnosis. On the other hand, the sensor's adhesion is optimized with respect to electrical‐force‐strain coupling and energy transfer efficiency at the interface between skin and sensor. The balance between the storage modulus and loss modulus are adjusted via the crosslinking degree of the polyacrylamide (PAAm) hydrogel network. As a result, the optimized knitted sensor enables stable collection of pulse waveforms from the radial and dorsalis pedis arteries. In human patient evaluations, the knitting‐based strain sensor can distinguish patients with different potential CVD risks through extracted characteristic indicators.

## Graphical abstract

This study focuses on the early diagnosis of CVDs through pulse waveforms, requiring wearable devices to achieve high‐fidelity monitoring of pulse waves from the body surface. Knitted strain sensors are optimized in terms of mechanical adaptation and adhesion capability. As a result, the optimized knitted sensors can stably collect pulse wave signals from the radial artery and dorsalis pedis artery, showing high consistency with waveforms recorded by clinical instruments.

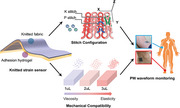

## 1 Introduction

CVDs are responsible for nearly 20 million deaths annually, making them the leading cause of death worldwide.^[^ ^1^ ^]^ Wearable devices offer critical early‐warning capabilities that can significantly reduce cardiovascular‐related mortality rates.^[^ ^2^ ^]^ Pulse waves (PW), which are low‐frequency mechanical waves originating from arterial deformation, are weak at the body surface but carry extensive physiological and pathological information related to the cardiovascular system.^[^ ^3^ ^]^ Continuous monitoring and accurate analysis of high‐fidelity PWs are essential for timely intervention in CVDs.^[^ ^4^, ^5^, ^6^, ^7^ ^]^ Textile‐based wearable devices offer advantages such as breathability and comfort,^[^ ^8^ ^]^ making them particularly suitable for the long‐term monitoring of elderly patients.^[^ ^9^ ^]^ Existing textile wearable sensors are predominantly applied for detecting large deformations, such as motion monitoring.^[^ ^10^, ^11^ ^]^ Some approaches have partially succeeded in detecting tiny signals like PWs and sounds by significantly increasing sensors’ sensitivity.^[^ ^12^ ^]^ However, sensors designed with low modulus for conformal skin contact often exhibit significant energy dissipation during deformation‐recovery processes.^[^ ^13^, ^14^ ^]^ This design results in prolonged recovery times, residual oscillation interference, which reduce detection accuracy and limited their ability to provide reliable pathological information. Additionally, these sensors are typically placed in concave skin regions, where common attachment methods, such as straps or wristbands, create gaps between the sensor and skin.^[^ ^15^, ^16^, ^17^ ^]^ These discontinuities at the interface lead to high mechanical impedance and low energy transfer efficiency, further exacerbating energy dissipation. Consequently, existing sensors face difficulties in collecting sufficiently accurate and detailed physiological mechanical signals.^[^ ^18^ ^]^

**Figure 1:**
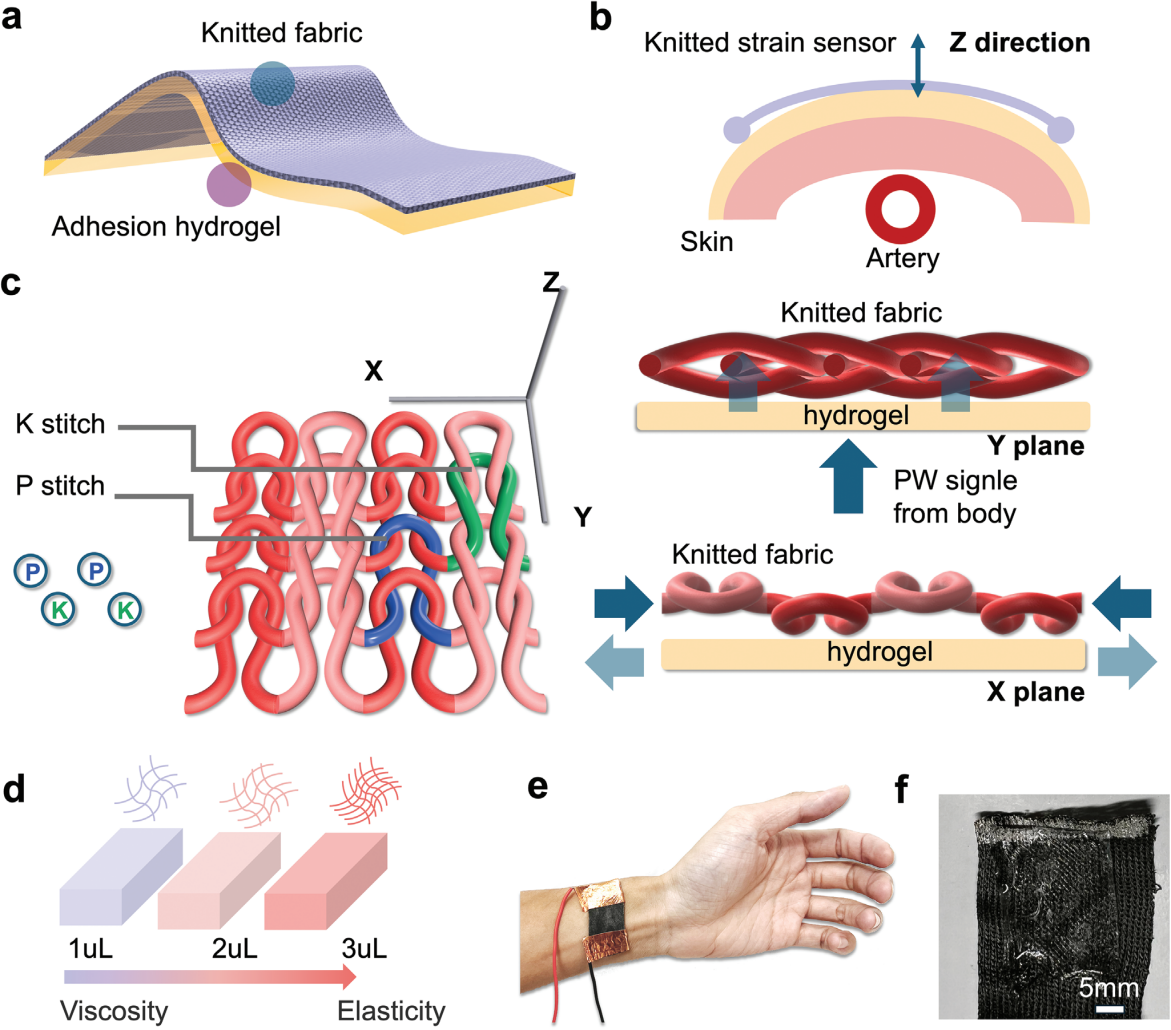
From knitted structures to the design of knitted sensors. a) A typical rib 1×1 knitted structure, with a front view illustrates the repeat units and side views in the X and Y directions. b) A flexible sensing structure composed of a knitted sensing layer and a hydrogel adhesive layer. c) Schematic representation of the sensing mechanism in the Z direction for the knitted bilayer sensor. d) Regulation of the stiffness of the adhesive layer by adjusting the concentration of the crosslinking agent. e) Application of the knitted bilayer sensor worn on the radial artery. f) Optical image of the knitted bilayer sensor.

Physiological mechanical signals, including PWs, are low‐energy longitudinal waves when transmitted to the skin surface.^[^ ^19^ ^]^ The deformation caused by these signals is complex, involving a combination of the sensor being constrained in the Y‐direction and subjected to forces in the Z‐direction (**Figure** **1**).^[^ ^20^ ^]^ However, most knitted sensors are calibrated using tensile tests in horizontal or vertical directions (defined as X and Y directions), respectively. These calibration methods cannot accurately represent the deformation experienced by sensors during PW detection. Therefore, it remains a challenge in how to reduce energy dissipation both within the knitted fabric and at the fabric‐skin interface, for collecting sufficiently accurate and detailed physiological mechanical signals.

To address these challenges, we optimized the knitted structure sensor^[^ ^20^ ^]^ and introduced an adhesive layer to minimize energy dissipation during signal acquisition (Figure 1a,b). The extent of slippage and deformation in the knitted fabric was controlled by adjusting the number and arrangement of knit (K) and purl (P) stitches (front and back stitches, respectively, in Figure 1c) in the X and Y directions.^[^ ^21^, ^22^ ^]^ By optimizing the loop arrangement within the knitted structure, we minimized the loss modulus during the deformation process, reducing stress dissipation during compress‐recovery cycles in the Z‐direction.^[^ ^23^ ^]^ Additionally, the crosslinking degree of hydrogels is positively correlated with their rigidity.^[^ ^24^ ^]^ We incorporated a hydrogel layer between the knitted sensor and the skin to enhance adhesion and coupling between the sensor and skin. By controlling the polymer network density of the hydrogel,^[^ ^25^, ^26^ ^]^ we tailored its viscoelastic properties to optimize energy transfer efficiency within the dynamic frequency range of PWs (0.5–2 Hz) (Figure 1d).^[^ ^18^, ^27^ ^]^

In this study, we utilized a knitted structure (Rib 11) in which adjacent and opposite loops exhibit symmetric positional relationships. This design achieved a balance between the storage modulus and loss modulus of the sensor in the Z‐direction.^[^ ^28^ ^]^ Simultaneously, by adjusting the hydrogel crosslinking degree, we ensured efficient energy transfer from PWs to the knitted sensor.^[^ ^10^, ^29^, ^30^, ^31^ ^]^ The optimized sensor successfully recorded high‐fidelity pulse wave signals from the skin surface (wrist and ankle). In clinical evaluations, the PWs recorded by this knitted sensor from elderly cardiovascular patients demonstrated predictive potential for assessing cardiovascular disease risk, offering a promising approach for wearable clinical diagnostics.

## 2 Results and Discussion

### 2.1 Design of Knitted Strain Sensor

Textile‐based wearable devices have shown unique potential among wearable electronics due to their superior comfort and long‐term wearability.^[^ ^32^, ^33^, ^34^, ^35^ ^]^ In recent years, significant advancements have been made in developing woven or knitted textile sensors for continuous monitoring of physiological signals, based on capacitive,^[^ ^36^, ^37^ ^]^ resistive,^[^ ^38^, ^39^ ^]^ and piezoelectric^[^ ^15^ ^]^ mechanisms. Knitted structures, characterized by their unique topology formed by stitched loops at an intermediate scale,^[^ ^40^ ^]^ possess qualities such as high breathability, lightness, softness, stretchability, and elasticity. These attributes, along with the potential for large‐scale production, have garnered increasing attention.^[^ ^41^ ^]^ Strain sensing in knitted fabrics is typically achieved through two methods: the first involves producing yarns from elastic substrates mixed with conductive materials, followed by knitting.^[^ ^42^ ^]^ This approach mainly relies on the increase in intrinsic resistance due to the stretching deformation of the yarn, leading to positive resistance changes. The second method uses conventional textile materials, such as wool and cotton, which are coated with strain‐sensitive conductive materials.^[^ ^43^ ^]^ In this case, the strain sensing is primarily achieved through changes in the bending degree of the yarn, leading to loop deformation and changes in contact point pressure, causing contact resistance variations on the surface of the yarn coated with few‐layered 2D nanomaterials, often resulting in negative resistance changes. However, knitted fabrics made from conductive elastic yarn also exhibit contact resistance changes at contact points during deformation. The opposing effects of these two mechanisms counteract each other, resulting in non‐linear strain‐force‐electric responses, which is particularly disadvantageous for small strain measurements. Consequently, non‐stretchable yarns coated with 2D materials have gradually become the mainstream choice in knitted strain sensors.

Applying strain sensors to detect PW from arterial pulsation, a typical mechanical physiological signal, the sensors adhered to the skin surface detect the small compression deformation (≤2%) of the skin. Due to this signal is too weak, it should take into considerations that the balance of viscoelasticity of the sensor during deformation by maximizing the ratio of the storage modulus to the loss modulus in the sensing material. Moreover, to minimize the ratio of storage modulus to the loss modulus in the adhesive material, the mechanical impedance of signal transmission to the sensor could be reduced. This approach enables more effective conversion of PW signals into energy that causes deformation of the strain sensor, thereby enhancing the sensor's sensitivity and signal‐to‐noise ratio, ultimately achieving high‐fidelity signal acquisition. Also, due to the relatively high bending‐to‐stretching stiffness, a one‐to‐many mapping relationship occurs between the dimensionless curvature and the bending stiffness ratio. This allows the knitted fabric to exhibit multiple stable configurations, enabling it to conform flexibly to any non‐zero Gaussian surface.^[^ ^44^, ^45^ ^]^

Based on these theories, we propose a dual‐layer strain sensor for high‐fidelity pulse wave monitoring on the skin surface. The sensor consists of a sensing layer made of knitted fabric coated with reduced graphene oxide (rGO) and an adhesive layer of PAAm hydrogel^[^ ^46^ ^]^ (Figure 1a). The sensor is extremely soft with high adhesive strength, allowing conformal attachment to complex curved surfaces of the skin (Figure 1e,f).

We designed both the sensing layer and the adhesive layer of the sensor. First, we recognized that in pulse wave measurement using knitted strain sensors, the loops in the knitted structure undergo both compression and slippage deformation modes, which differs from the traditional calibration modes of tensile and compressive sensors. As the knitted structure deforms, the relative positions of the loops change, leading to slippage between contact points. The presence of friction results in significant energy dissipation, causing nonlinearity in the stress‐strain relationship during stretching and recovery, which affects the sensor's fidelity. We found that by adjusting the knitted structure, it is possible to alter the arrangement of P stitches and K stitches within a row of loops in the fabric (Figure 1c; Figure S1, Supporting Information). In the rib 1×1 structure, P and K stitches are alternately arranged, which minimizes friction between loops. Additionally, based on our previous research, we selected the 14G machine gauge and 2 stands parameter for knitting fabrication (Figure S2, Supporting Information). Compared to previous designs,^[^ ^20^ ^]^ this looser fabric structure resulted in a lower modulus and more deformation between contact points, further reducing energy dissipation.

For the adhesive layer, high adhesive energy requires effective energy dissipation when the interface is subjected to stress. This energy dissipation is achieved through hysteresis in the adhesive or the substrate.^[^ ^47^ ^]^ However, this conflicts with the requirement to transmit the PW signal to the sensor with high precision and without delay. To address this issue, we modified the viscoelasticity of the PAAm hydrogel by varying the amount of crosslinker added.^[^ ^46^ ^]^ The amount of crosslinker alters the density of the hydrogel's molecular network: a higher amount increases elasticity and decreases viscosity. However, when viscosity is high and elasticity is low, energy dissipation is greater, whereas the opposite condition results in more energy storage.^[^ ^48^ ^]^ Therefore, we optimized the crosslinker concentration to match the mechanical impedance for pulse wave propagation speed and frequency.

### 2.2 Fabrication and Characterization of Knitting Strain Sensor

**Figure 2:**
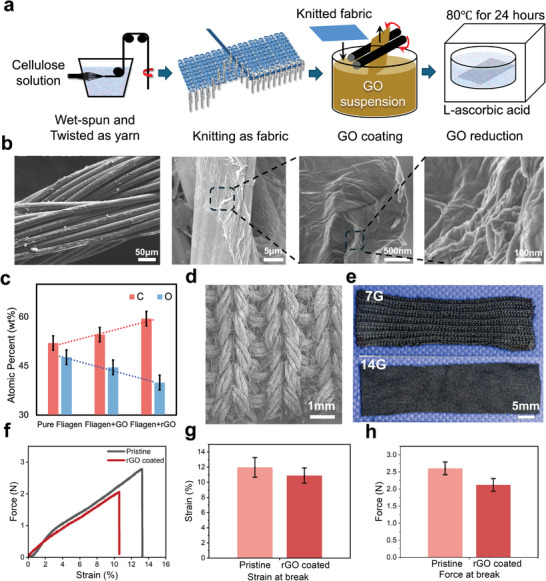
Preparation and characterization of knitted strain sensors. a) The fabrication process of knitted strain sensors. b) The SEM images from different scales to present how rGO coated onto the surface of yarns and fibres. c) The EDX results of pristine yarns, GO coated yarns and rGO coated yarns. d) The SEM image of knitted structures. e) Optical image of two gauges of knitted sensing fabrics. f) The force‐strain curves for pristine yarns and rGO coated yarns. g) Force at break and h) Strain at break of these two yarns.

Based on previous studies, we fabricated knitted strain sensors via a GO pad‐dry and in situ reduction technique (**Figure** **2**a). We first employed yarns produced through a collagen and cellulose blend process to fabricate knitted strain sensors. The yarn was knitted using a digital flatbed knitting machine. The pristine knitted fabrics were coated by immersing and rolling them in GO aqueous solution (5 mg mL^−1^), with the process repeated three times. The GO‐coated fabrics were then placed in a hydrothermal autoclave containing ascorbic acid solution (50 mg mL^−1^), heated to 80 °C, and maintained for 24 hours.

SEM resluts revealed that the microstructure of the knitted fabrics remained intact, with fibers uniformly covered by multiple layers of rGO. The rGO surface exhibited abundant wrinkles (Figure 2b). EDX results indicated an increase in the proportion of carbon (C) and a decrease in oxygen (O) on the fabric surface post‐coating and reduction (Figure 2c; Figures S3, S4, Supporting Information). Raman spectroscopy (Figure S5, Supporting Information) and water contact angle measurements (Figure S6, Supporting Information) further supported the successful in situ reduction of GO on the fabric surface. After reduction, the D/G ratio increased from 0.85 to 1.06 and the carbon (C) ratio on the fabric surface increased from 52% to 65%. Both of those indicate that the functional groups on the fabric surface are removed, leaving only rGO composed solely of carbon atoms, indicating that the GO on the fabric surface has been fully reduced.

We use 2 gauges of knitting machine to prepare the knitted fabrics for comparison, 7G and 14G (Figure 2e), where gauge number represents the number of loops per inch in the fabric. The 7G fabric had a rougher texture and more flexible, loose loops, while the 14G fabric, though thinner, had tighter loops, making it stiffer (Figure 2d). Also, tensile mechanical tests on pristine and GO‐coated yarns showed that rGO‐coated yarns had slightly reduced mechanical properties but retained a similar Young's modulus. The yarns exhibited a higher tensile modulus and lower elastic deformation range compared to the fabrics (Figure 2f–h).

### 2.3 Regulating Sensor Loss Modulus by Different Kinds of Knitted Structures

**Figure 3:**
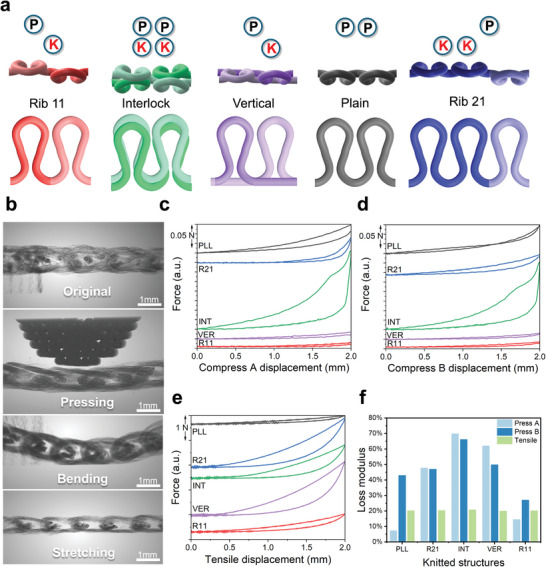
The loss modulus study on five kinds of knitted structures. a) The morphologies and structures of different types of knitted fabrics. b) The CT images from a side‐view for a knitted fabric (Rib 11) at its original, under a pressing, under a bending and under a stretching. Loop hysteresis of different knitted fabrics under a pressing from side A c), side B d), and under a tensile e). f) Loss modulus of different knitted fabrics.

To enhance the sensing performance of knitted strain sensors, precise control of the loop “intermediate scale” size and relative position is essential. This study introduces two sizes and five different fabric structures for strain sensor fabrication, encompassing most loop arrangements achievable with current knitting techniques. **Figures** **3**a and S1 (Supporting Information) present the minimal repeating units of the five structures. Loops resemble omega (Ω) shapes, curving in the same direction as the knitting needles. Plain structures use one side of the needles, while other structures use two sets of needles. In Plain (PLL), Rib 11 (R11), and Rib 21 (R21) knitted structures, a single yarn is used, whereas Interlock (INT) and Vertical (VER) structures use two yarns. The difference between Vertical and Interlock lies in the needle arrangement; Vertical structures knit on one side, while Interlock structures have face‐to‐face loops, forming a double‐layer structure. Using CT imaging (Figure 3b), we analyzed in situ deformation of knitted fabrics under pressing, bending, and stretching conditions, revealing distinct loop deformations compared to the fabric's original state. When measuring subcutaneous pulse signals, a small portion of the sensor experiences pressing deformation, with loops compressed at the point of pressure and slightly stretched elsewhere. Bending the sensor causes loops to become shorter and fatter, while stretching causes loops to become elongate.

From the in situ micro‐CT results, we have understood that when a knitted strain sensor functions as a wearable device for PW monitoring, it detects the mechanical signal through a pressing mechanism. In this context, it is essential to simulate the mechanical and electrical synchronous characterization techniques and emulate the reception of the pulse wave signal from the subcutaneous tissues. Therefore, we designed and fabricated a custom mechanical testing kit via metal 3D printing, compatible with a universal testing machine. Considering that the front and back sides of certain knitted structures are not symmetrical, we measured the hysteresis loops between force and deformation for five different fabrics during compression on side A (Figure 3c; Figures S7, S8, Supporting Information), side B (Figure 3d), tension (Figure 3e; Figure S9, Supporting Information), and expansion (Figure S10, Supporting Information). Subsequently, for each type of fabric and each form of deformation, we calculated the energy during both the deformation and recovery phases, with the ratio of these energies being regarded as a measure of energy dissipation, also referred to as the dissipation modulus. The results (Figure 3f) indicate that the dissipation modulus for various fabrics under tension is relatively consistent, around 20%. However, there is significant variation in the dissipation modulus during compression. Overall, the dissipation modulus under compression is lower than that under tension for the Plain and Rib 11 fabrics.

### 2.4 Regulating of Hydrogel Modulus and Adhesion Capability

**Figure 4:**
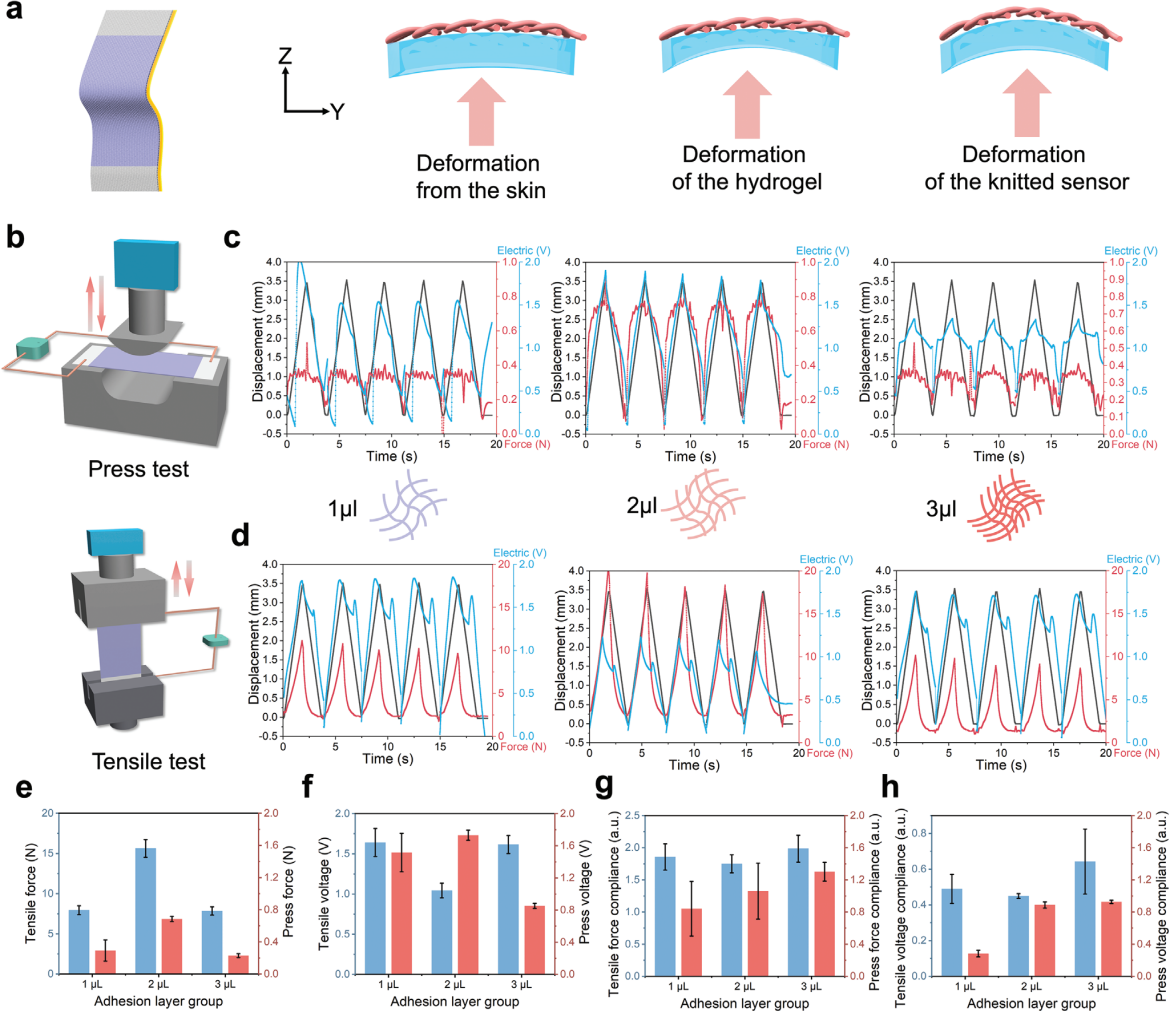
The electric‐force‐deformation coupling performances of knitted sensor with adhesive layer. a) Schematic diagram of sensor deformation under a signal from Z direction. b) Schematic diagram for press and tensile test. The deformation‐force‐electric coupling for sensor with adhesive layer in different crosslink degree under a pressing c) and tensile d). The range of force e) and voltage (e) under a 10% strain deformation, and their compliance g, h).

The fabric itself does not possess adhesive properties. Although some previous studies have used wristbands to secure knitted sensors,^[^ ^49^ ^]^ this method does not ensure mechanical impedance matching between the sensor and the skin. This can lead to slippage and friction between the fabric and the skin, resulting in energy dissipation, which further reduces the signal‐to‐noise ratio and exacerbates signal distortion. To address this issue, we placed a layer of PAAm hydrogel with a controlled thickness on the surface of the knitted sensor via in situ crosslinking. By simply adjusting the amount of crosslinking agent, we were able to modulate the modulus of the gel to achieve suitable mechanical impedance matching (**Figure** **4**a). When the knitted sensor adheres to the skin through the gel, the deformation of the skin is first transmitted to the gel and then to the fabric. Therefore, minimizing the attenuation and distortion of the signal by the gel is crucial. The modulus of the hydrogel is primarily regulated by the density of its crosslinking network. For PAAm hydrogel, the amount of tetramethyl‐ethylenediamine (TEMED) added is key to controlling the degree of crosslinking. When a small amount of TEMED is added, the viscous component of the hydrogel is dominant, leading to a high loss modulus and a low storage modulus. In this case, the energy in the PW signal is dissipated in the viscous deformation of the gel, making it difficult to transfer to the fabric. Conversely, when an excessive amount of TEMED is added, the elastic component of the hydrogel dominates, resulting in a low loss modulus and a high storage modulus. In this scenario, the energy in the PW signal is stored in the elastic deformation of the gel. Considering the continuous periodic nature of PW signals, the delayed energy transfer can lead to signal misalignment and distortion. For PW signals with a narrow frequency fluctuation range, there exists an optimal degree of crosslinking that balances the loss modulus and storage modulus.

We demonstrated the force‐strain‐electrical signals (Figure 4b) of the bilayer sensor under tensile (Figure 4c) and compressive (Figure 4d) loading after adding 1 µL, 2 µL, and 3 µL of TEMED crosslinking agent to 0.6 mL of hydrogel precursor solution, which is mixed in proportion of acrylamide, N,N’‐methylenebis (acrylamide) (MBAA), and ammonium persulfate (APS).

**Figure 5:**
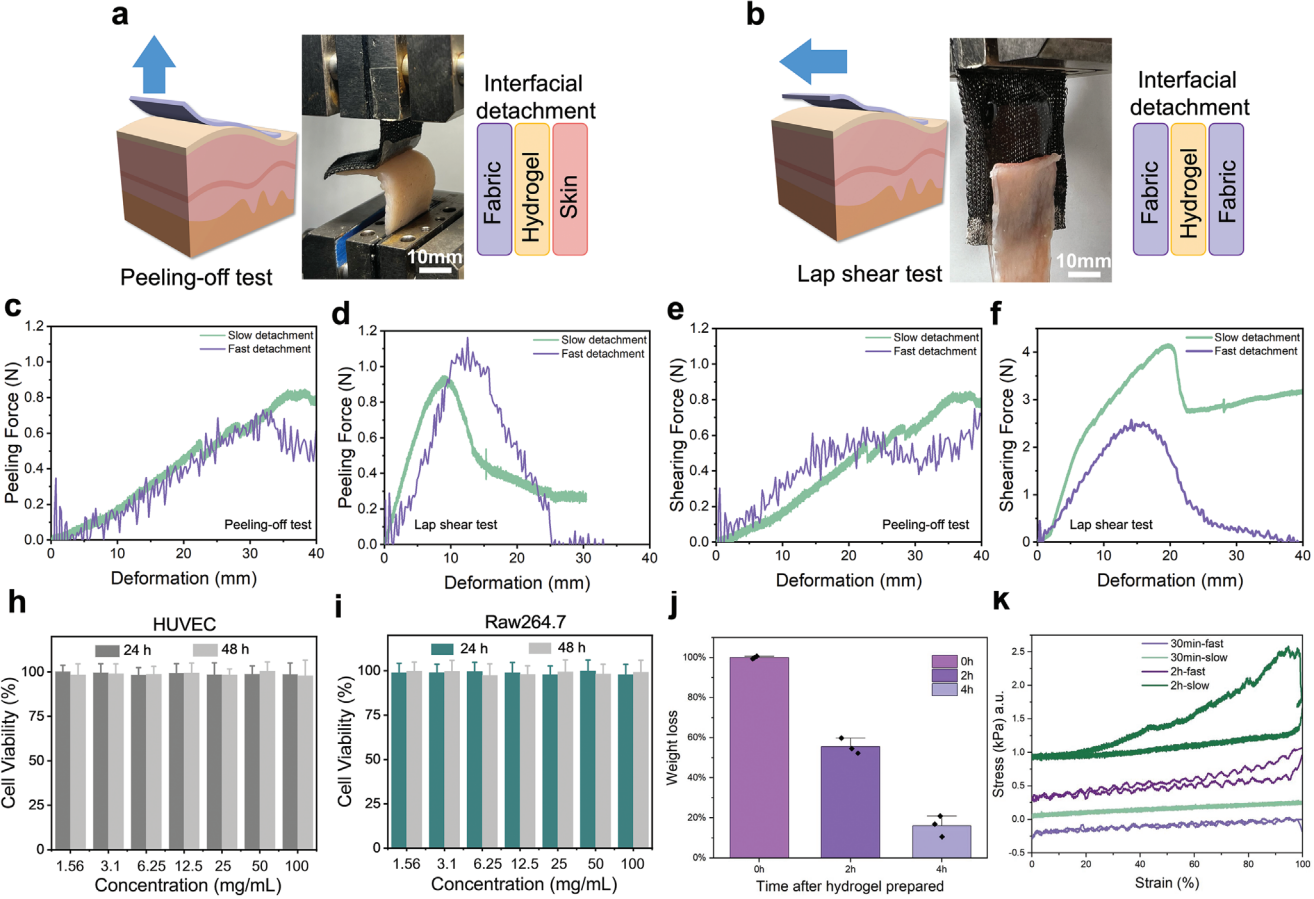
The adhesion, biocompatibility and durability studies of bilayer sensor. a) Schematic diagram and photo of peeling‐off test. b) Schematic diagram and photo of lap shear test. c,d) Test for exfoliation from porcine skin. e,f) Test for exfoliation from knitted fabric. h,i) Biocompatibility based on HUVEC endothelial cells and Raw264.7 macrophage cells co‐culture with the PAAm hydrogel. j–i) The weight loss rate of PAAm hydrogel and its tensile behaviour.

The result of attenuated total reflectance Fourier‐transform infrared (ART‐FTIR, Figure S11, Supporting Information) of PAAm hydrogel indicates the presence of a prominent C═O stretching band (amide I) and an N─H bending band (amide II) in the hydrogel network. The relationship between the mechanical properties and crosslinking density of hydrogels was established through small‐angle X‐ray scattering (SAXS, Figure S12, Supporting Information), differential scanning calorimetry (DSC, Figure S13, Supporting Information), and mechanical modulus tests (Figure 5k). The series of characterization results evaluated the impact of TEMED on crosslinking. DSC data suggests that the addition of TEMED enhances the crosslinking density within the hydrogel, leading to an increased polymer network. The SAXS results for the 2 µL group show more significant scattering in the low‐q region, indicating phase separation induced by hydrogen bonding interactions and the formation of large aggregates. These findings are consistent with the improved mechanical properties of the 2 µL group.

From the tensile tests, we found that the bilayer sample with 2 µL of TEMED exhibited the highest tensile modulus. In contrast, the 3 µL sample showed poor synchronous stretching of the PAAm hydrogel with the fabric fixed in the clamps during tensile testing, likely due to the higher degree of crosslinking, which increased the modulus. In the compression test, the PAAm hydrogel contacts with the test machine, which results in the force and deformation are transmitted through the gel to the fabric and cause an electrical response. The results reveal that the electrical response of the 1 µL sample exhibits significant hysteresis, attributed to insufficient crosslinking leading to excessive viscosity of the material. Conversely, the 3 µL sample displays regular oscillations upon recovery, which is due to excessive crosslinking resulting in high material elasticity. Notably, the force measured for the 3 µL sample during compression is lower than that of the 2 µL sample; however, it exhibits a longer plateau phase of sustained force. This phenomenon can be explained by the positioning of the force measurement device close to the gel side, indicating that highly crosslinked gels have a higher storage modulus, which hinders the transmission of mechanical signals. Tensile testing corroborates this observation, revealing that the 2 µL sample has the highest tensile modulus. It was also observed that during stretching, the PAAm hydrogel in the 3 µL sample did not stretch synchronously with the fabric, which was fixed at both ends in the grips. Consequently, although the 3 µL sample theoretically possesses a higher modulus, its adhesion capability is insufficient. Therefore, the 2 µL sample represents the optimal balance between adhesion ability and mechanical impedance matching, making it the best choice for use as an adhesive layer in PW body surface monitoring sensors (Figure 4e–h).

For knitted sensing layers, the PW signal is influenced by its composite deformation in the Y and Z directions. Therefore, for the PAAm hydrogel adhesive layer, it is essential to ensure high‐strength adhesion to both the fabric and the skin while still allowing the PW signal to pass through. In this study, we designed 90° and 180° exfoliation tests to evaluate the adhesion capability of the hydrogel (**Figure** **5**a,b). Here, we used PAAm hydrogel with the addition of 2 µl TEMED crosslinker. The adhesion capability of PAAm hydrogel is significantly higher than the force exerted on the knitted fabric under the same deformation, which demonstrate sufficient adhesive stability. The PAAm hydrogel exhibited stronger adhesion to the knitted fabric than the porcine skin, which may be attributed to the partial penetration of the PAAm hydrogel solution into the fabric during in situ crosslinking of the gel on the fabric surface. We demonstrated two rates (1 mm ^−1^s for fast detachment and 0.1 mm ^−1^s for slow detachment) of exfoliation tests, both at peeling‐off test and lap shear test, from porcine skin (Figure 5c,d) and our knitted fabric (Rib 11, Figure 5e,f). The results showed that the peeling rate had a smaller impact on the 90° test, especially on skin tissue. During peeling‐off test, the relationship between force and tensile displacement was relatively linear. In the lap shear test, the PAAm hydrogel exhibited stronger adhesion to porcine skin and knitted fabric compared to the peeling‐off test and had a higher modulus. Although fast detachment reduced the modulus at low strain stages, the adhesive strength remained sufficient.

To assess the potential harm that hydrogel adhesive layers may cause during prolonged wear and removal, we conducted testes on cells and human volunteers. In the cell experiments, we selected two cell lines commonly found in skin tissue: HUVEC endothelial cells and Raw264.7 macrophage cells, for co‐culture with the hydrogel.^[^ ^50^ ^]^ After 24 and 48 hours of culture, CCK‐8 assays were performed. The results (Figure 5h,i) showed that both cell lines exhibited desired viability after 48 hours of co‐culture (97.89% for HUVEC and 97.35% for Raw264.7), indicating the biocompatibility and low cytotoxicity of the PAAm hydrogel. This suggests that the interaction between the PAAm hydrogel and skin cells does not cause significant cell damage, making it suitable for use as the adhesive layer in sensors. Subsequently, we conducted a 24‐hour wearability test of the sensor on the dorsum of a volunteer's foot. The results demonstrated that the sensor remained safe, secure, and effective wearability over the 24‐hour period. Based on these experimental findings, we proved that the PAAm hydrogel meets the mechanical compatibility, biocompatibility, and robustness requirements for adhesive layers in long‐term wear applications.

To further assess the hydrogel's durability, we prepared a 50 mm diameter hydrogel from 3.5 mL of hydrogel solution, which was fixed and stretched tightly over a square frame with a diameter of 75 mm. The sample was placed it in a fume hood, and its weight loss was recorded over time. The results (Figure 5j) indicated that the hydrogel's weight decreased by approximately 40% after 2 hours, at which point it still maintained good adhesion and shape recovery capabilities, with minimal difference from its initial state. However, after an additional 2 hours, the hydrogel had completely hardened and lost its adhesive and mechanical properties. It is important to note that the rate of dehydration and evaporation of hydrogel depends on environment and surface area, and the experimental setup used here is more extreme than typical usage conditions. Based on the weight loss experiments, we fabricated hydrogel strip samples measuring 2×2×10 mm for mechanical tensile testing. The tensile region was the central 50 mm‐length part of the hydrogel samples, with a strain range of 0–100%. We selected two loading rates: 1 mm ^−1^s and 0.1 mm ^−1^s. The tensile‐recovery behavior tests were conducted after 30 min and 2 h of hydrogel preparation. The results (Figure 5k) indicated that after undergoing large deformation stretching and prolonged drying, the hydrogel retains its modulus during tensile recovery at a higher speed (which is similar to the absolute rate of deformation during PW measurement) even in a dehydrated state.

### 2.5 Quantitative Sensor Performance Evaluation toward PW Monitoring

**Figure 6:**
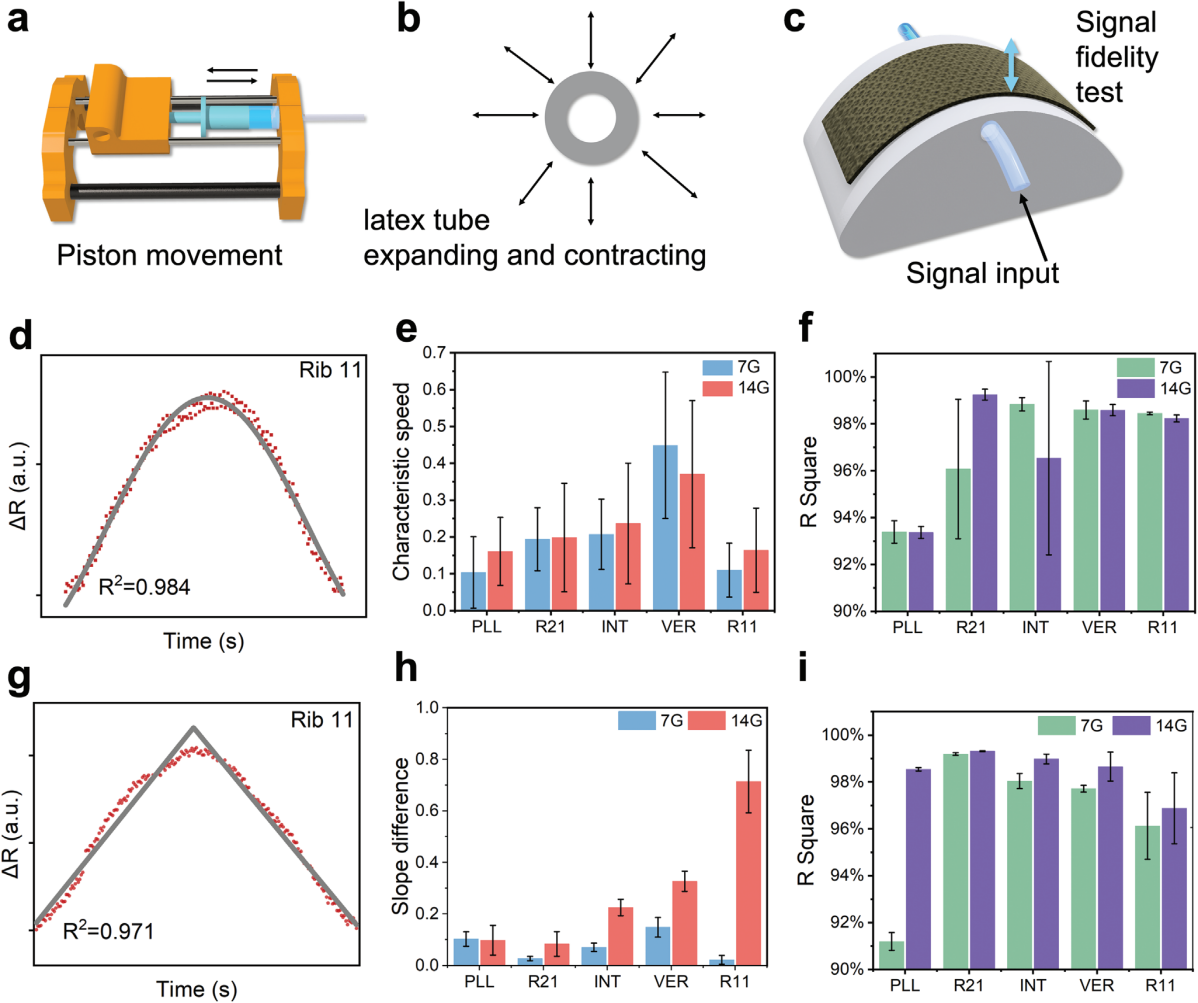
Signal fidelity test for knitted strain sensors with different gauges and structures. a, b) Schematic of z‐direction signal generator with controllable latex tube expanding and contracting. c) Schematic of signal fidelity test by using a customized device. The sensing performance of each knitted strain sensor to present a linear reciprocating triangular wave signals and half‐sine wave signals. d) Half‐sine waveform fitting results for knitting strain sensor. e) The characteristic speed of press and recovery. f) The fitting R^2^ value for half‐sine wave forms from each knitted sensor. g) Triangular waveform fitting results for knitting strain sensor. h) The slope difference of press and recovery. i) The fitting R^2^ value for triangle wave forms from each knitted sensor.

Taking the pulse signal as an example, the mechanical waves generated by physical deformation within the human body propagate outward through subcutaneous tissues and skin before reaching the body surface. A sensor affixed to the skin deforms under the influence of these mechanical waves. Therefore, obtaining high‐fidelity signals hinges on the sensor's accurate and linear detection of strain in the Z‐direction. To simulate the environment and conditions where the fabric sensors adhere on the skin surface and captures mechanical signals originating from subcutaneous tissues or deeper sources, we designed a device to mimic arterial deformation. In brief, this device comprises a linear motion module driven by a stepper motor, which moves a water‐filled piston connected to a latex tube. One end connected to the piston, while the other end sealed. The motor's forward and backward movements cause the latex tube to expand and contract (**Figure** **6**a; Figures S15, S16, Supporting Information). To further emulate the embedding of superficial arteries under the skin, we cast a curved surface using Ecoflex (Figure 6b). The motor‐driven piston movement provides programmable, periodic reciprocating motion, ensuring consistent mechanical signal amplitude delivered to the latex tube. This design avoids high‐frequency vibrations typically generated by impeller pumps, thereby maintaining signal purity.

Using this setup, we generated half‐sine wave signals and linear reciprocating triangular wave signals to more comprehensively evaluate the signal fidelity of sensors with different fabric structures. The triangular wave primarily assesses the sensor's linearity and response to abrupt stops and starts, while the sine wave, with its continuously varying curvature, mainly evaluates the sensor's accuracy. Subsequently, the obtained signals were split by cycle, baseline‐corrected along the X‐axis, and amplitude‐normalized along the Y‐axis. Signal processing was conducted using MATLAB, and the results are shown in Figure 6c–f and Figure S14 (Supporting Information).

We fitted the triangular wave function using two linear segments (Figure 6g). Besides the fitting degree, the difference in the absolute values of the slopes of the two linear segments represents the sensor's differential response during compression and recovery. A smaller difference indicates less energy dissipation during sensing by the knitted structure, providing higher consistency. The results show that the Plain and Rib 21 structures, which have asymmetric tension, exhibit higher recovery slopes, indicating a faster recovery rate than the compression rate. This further demonstrates that the inward curling force of the fabric can affect sensing performance. Interestingly, the Vertical structure also exhibited a faster recovery rate, possibly due to the participation of unlooped yarns in the deformation. The Interlock and Rib 11 structures exhibited slower recovery rates, with the Rib 11 structure in 7G showing the smallest difference. We believe that the alternating front‐back loop arrangement of Rib 11 and the moderate loop size of the 7G gauge ensure consistency between compression and recovery.

Further, we used nonlinear fitting to assess the fidelity of the ten different knitted strain sensor structures in measuring sine waves. The fitting degree of the function indicates the similarity between the input and output waveforms (Figure S15, Supporting Information). All ten sensor structures demonstrated high fitting degrees. Comparatively, the 14G fabric sensors exhibited better fitting degrees than the 7G samples, likely due to the smaller loop size of the 14G fabrics, which is closer in scale to the superficial arteries. Among the 14G sensors, the fitting degrees of the Plain, R21, and R11 structures were significantly higher than those of the INT and VER structures. We believe that the double‐layer structure of INT and the unlooped transverse yarns in VER may obstruct and delay the continuous and gentle strain‐recovery process, thereby affecting the waveform fidelity. For the better‐performing Plain, R21, and R11 structures, the R11 structure exhibited high fidelity in both strain and recovery phases, while the Plain and R21 structures showed more linear and steep waveforms. This is because the asymmetrical structures of PLL and R21, where PLL has only front‐facing loops and R21 has two rows of front‐facing loops and one row of back‐facing loops, create uneven tension across the fabric, causing it to curl toward the lower tension side. Contacting the skin with the lower tension side can enhance adhesion and improve sensing performance. However, this tension is challenging to control, leading to some distortion in the four asymmetric structures studied in this research.

### 2.6 Evaluation the Vascular Health via Knitted Sensor Acquired Pulse Waveforms

**Figure 7:**
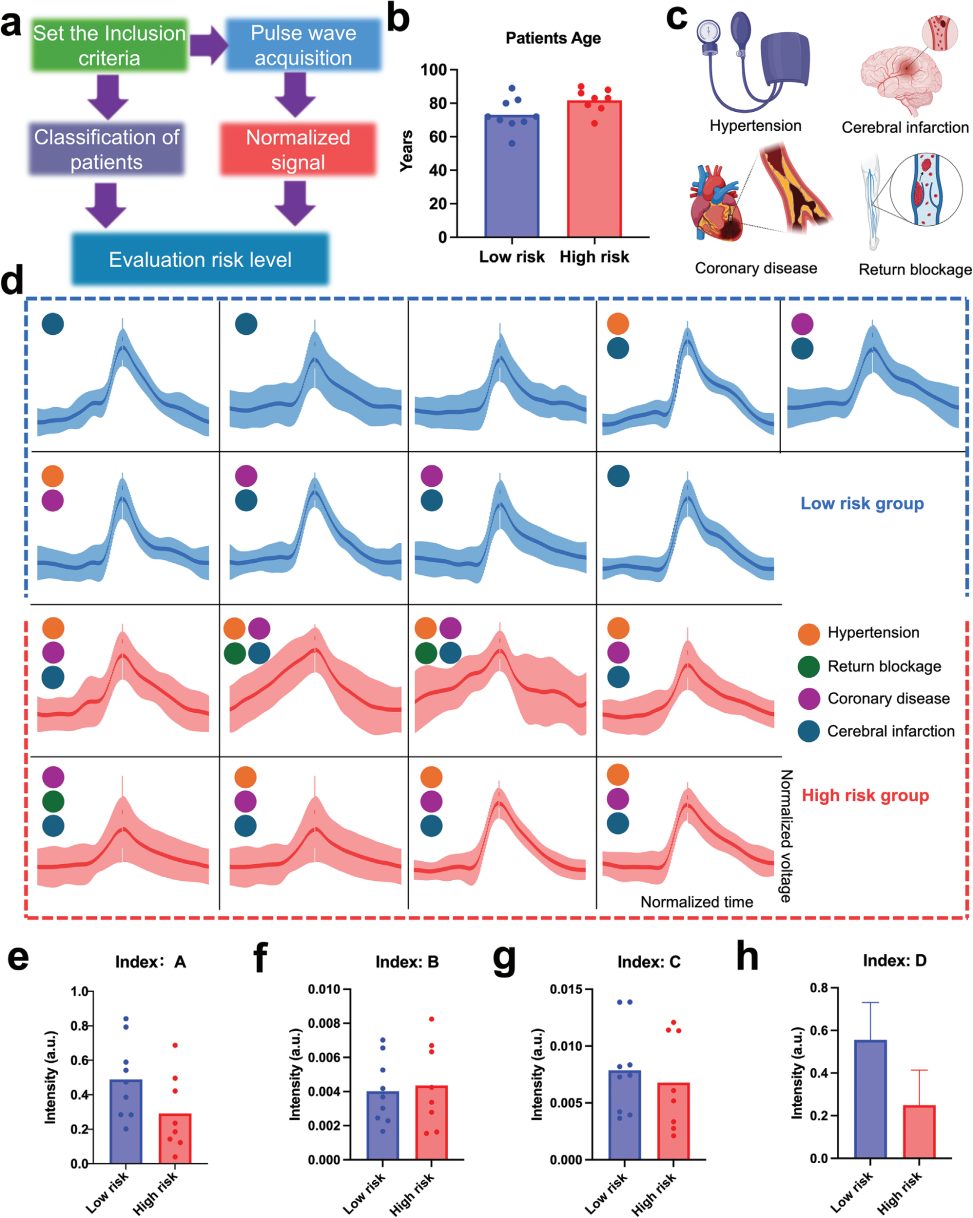
Evaluation the vascular health via knitted sensor acquired pulse waveforms. a) Flowchart of data process of the clinal test. b) The age distribution of 17 enlisted volunteers. c) The inclusion criteria of this test. d) The normalization pulse waveforms of each volunteer and e–h) extracted 4 indicators.

We introduced a strain sensor with dual modulus adaptation, based on knitted structures and the degree of hydrogel crosslinking, into clinical trials to assess patients’ vascular health by detecting the pulse waveforms from their feet (**Figure** **7**a). Our attempt contributes to pulse wave‐based diagnosis of clinical vascular diseases. Pulse wave data was collected from the feet of 17 patients hospitalized in a department of geriatrics. These 17 patients, aged between 56 and 90 (Figure 7b), all of whom had varying degrees of chronic underlying diseases. Among them, four typical degenerative vascular diseases—cerebral infarction, coronary atherosclerotic heart disease, grade 3 hypertension, and lower limb venous reflux disorder—were used as criteria for patient admission, and the patients were classified into two groups. Patients diagnosed with all four or three of these diseases were classified as the “extremely high‐risk group,” while those with only 1–2 of these diseases were classified as the “moderately high‐risk group.” This classification is due to the fact that the co‐occurrence of these four diseases indicates that the patient is in a very poor state regarding vascular stiffness, hemodynamic, and the extent and distribution of emboli (Figure 7c), with the risk of danger at any time. Patients with only 1–2 diseases have a lower risk. Among the groups, 8 elderly patients met the criteria for the “high‐risk group”, and 9 patients met the criteria for the “low‐risk group”. We aimed to differentiate these two groups based on the pulse waveforms recorded by the knitted sensors. To achieve this, we normalized the data recorded from each patient's feet (Figure 7d) and then extracted four indicators: Indicator A: the slope of the ascending segment, Indicator B: the integral of the ascending segment, Indicator C: the integral of the descending segment, Indicator D: the presence or absence of a dicrotic notch in the descending segment, and Indicator E: the presence or absence of an inflection point before the ascending segment.

For Indicator A (the slope of the ascending segment), it represents the rate of vascular expansion. Since heart pumping is a rapid process, a normal pulse wave has a steep ascending segment. A gentler slope indicates lower vascular compliance, while a steeper slope may suggest vascular blockage. For Indicator B (the integral of the ascending segment), it also reflects the rate of vascular expansion during a rapid pumping process, a lower integral value is expected, whereas a higher value suggests vascular blockage. For Indicator C (the integral of the descending segment), it represents the spatio‐temporal distribution of the vessel during the expansion phase. A higher integral value indicates that the vessel slowly decreases to diastolic pressure as blood pressure drops, reflecting poor elasticity and a higher vascular modulus. Conversely, a lower value indicates better vessel compliance and elasticity. Indicator D (presence of a dicrotic notch in the descending segment) represents an echo caused by blood flow striking the vessel wall after mitral valve closure. The presence of a dicrotic notch indicates better vessel elasticity and lower peripheral resistance, while the opposite suggests venous or capillary reflux obstruction.

In the results, we found that compared to the “low‐risk group,” Indicator A was higher in the “high‐risk group” (Figure 7e), Indicator B was lower (Figure 7f), Indicator C was higher (Figure 7g), and Indicator D was more pronounced (Figure 7h). These findings align with the physiological implications of the indicators. Through the experiments, we discovered that the pulse wave sensor, with dual modulus adaptation based on knitted structures and hydrogel crosslinking, can record high‐fidelity pulse waveforms in geriatric patients with multiple comorbidities. From these waveforms, we were able to extract characteristic indicators related to vascular hypertension, stiffness, embolism, and reflux obstruction, enabling differentiation of disease severity among patients. This capability facilitates a comprehensive cardiovascular assessment of elderly patients, with potential applications in long‐term monitoring, home care, and chronic disease management.

## 3 Conclusion

This work elucidates that the energy dissipation and force‐deformation‐electrical coupling mechanisms of knitted mechanical sensors in response to Z‐direction signals differ significantly from those observed during stretching. Building on this understanding, we propose an energy dissipation strategy that specifically modulates the dissipative modulus and storage modulus of the knitted fabric and PAAm hydrogel, thereby enhancing the overall mechanical reliability of the sensor. Through dual regulation of the knitted structure and the crosslinking degree of the PAAm hydrogel, we significantly improved the sensor's matching to the PW‐specific mechanical impedance, validating its effectiveness in capturing high‐fidelity PW signals for clinical diagnostics. The knitted sensors, integrated with the hydrogel adhesive layer, demonstrated the ability to stably extract human PW signals, with characteristic values comparable to those obtained from bedside monitoring equipment. This sensor fabrication concept holds promise for impacting the extraction of weak physiological mechanical signals from the body surface.

## 4 Experimental Section

### Knitted Fabric Fabrication

Collagen/cellulose yarns (FILAGEN, RCT New Materials Co. Ltd, PR. China) were used as received. These yarns were produced from a mixture of collagen peptide and viscose solution via the wet‐spinning method, with a final fiber count of 40s. The yarn used for knitting consisted of two 40s fibers. The width of the knitted fabrics was 5 cm. Five types of knitted structure fabrics were produced using 7G (gauge) and 14G (gauge) machines, including plain, 2 × 1 rib (Rib 21), interlock (INT), ribbed vertical stripe (Vertical), and 1 × 1 rib (Rib 11). Ultimately, 10 structures of collagen/cellulose knitted fabric were prepared for further functionalization.

GO dispersion was prepared using expanded graphite flakes (+50 mesh flake size, Sigma‐Aldrich, USA) based on an optimized Hummers’ method. The GO was then diluted in deionized water at a concentration of 5 mg mL^−1^. The pad‐dyeing method was employed for GO coating. For each batch, the fabrics were padded with three cycles of coating, drying in an oven at 40 °C for approximately 10 minutes after each cycle until completely dry. After coating, the GO was reduced by immersing the fabrics in an aqueous solution of L‐ascorbic acid (L‐AA, Sigma‐Aldrich, MO USA) (50 mg mL^−1^) at 80 °C for 24 hours. A scanning electron microscope (SEM) (FEI Quanta 650 FEG, MA USA), followed by energy‐dispersive X‐ray spectroscopy (EDX), was used to analyze the atomic content on the surface.

### PAAm Hydrogel Preparation

The mixture of 20 mL of acrylamide solution (12% w/w), 144 µL of MBAA (2% w/w), and 452 µL of APS (66 mg mL^−1^) was stirred until fully dissolved. The pH of the PAAm hydrogel was adjusted to the desired level by adding 1 M NaOH or 1 M HCl solution to the acrylamide precursor solution. The solution was then left to rest overnight to remove any bubbles. A 0.6 mL aliquot of the acrylamide precursor solution was placed into a mold with a removable bottom partition (30 mm × 15 mm), with knitted fabric positioned underneath the mold. Next, 1 µL, 2 µL, or 3 µL of TEMED crosslinker was added and quickly stirred. The setup was then exposed to high‐intensity light (500 lm) for 15 seconds. The partition was removed, allowing the semi‐crosslinked PAAm hydrogel to contact with the knitted fabric, followed by another 60‐second rest period to ensure full gelation of the hydrogel.

### Knitted Strain Sensor Preparation

For the sensing layer, after preparing and coating the fabric with rGO, the material was cut into dimensions of 30 mm × 15 mm, with top and bottom areas measuring 5 mm × 15 mm. Silver paint (ELECTROLUBE, UK) was applied as electrodes. For the adhesive layer, the PAAm/MBAA/APS hydrogel crosslinked by TEMED was peeled off from template and attached to the knitted fabric.

### Knitted Structure Morphology In Situ Observation

The morphology of the knitted sensors during deformation was investigated using nanoscale 3D X‐ray microscope computed tomography (CT) images (Carl Zeiss, Germany). Five types of knitted fabrics were mounted onto a custom‐designed tool capable of precise stretching, bending, and pressing. Images were taken for each knitted fabric in its original, stretched, bent, and pressed states. These images were then reconstructed into 3D models using Dragonfly software.

### Sensor Electrical‐Mechanical Test

The top pressure deformation in the Z‐direction was conducted with the aid of a stainless‐steel 3D printed model. In brief, an optimized three‐point bending method was used, where the two sides of the fabric were fixed to the model with a 10 mm overhang in the middle, and a curved top‐pressing block was attached to a force sensor and moved downwards to achieve top‐pressing.

A constant high‐precision current source was supplied by a source meter (Keithley 2612B, OH USA) to the conductive knitting fabrics. The electro‐mechanical performance of the weft knitting fabrics was quantitatively investigated using a combination of a mechanical test system (MTS Criterion Model 42, MN USA) with a 10 N load cell and an oscilloscope with a 160 Hz sampling frequency (DIGILENT Analog Discovery 2, WA USA). For each knitting structure, the test was repeated 30 times to obtain the resistance changes for the entire knitting sensor.

To further evaluate properties related to the detected pulse wave (PW), a specialized device to simulate PW generation was designed. This device included a microcontroller (ATmega328P, PR. China) to generate various wave types. The wave output was applied to a stepper motor (Tb6600 drive, PR. China) to pump water into dead‐end tubing, with the wave input functions including a half‐sine wave and a trapezoidal wave. The knitting sensor was mounted on an Ecoflex sponge covering the dead‐end tubing to mimic human skin tissue.

### Cell Viability Test

HUVECs and RAW264.7 (Institute for Biological Sciences, Shanghai, PR. China) were cultured in conventional conditions. Cell viability of HUVECs were measured using CCK‐8 assay (Beyotime Biotechnology, Shanghai, PR. China). Briefly, to evaluate cytotoxicity, HUVECs were seeded at 1×10^4^ cells per well and cultured with PAAm hydrogel (1.56‐100 mg mL^−1^) for 24 h and 48 h. Cells were also seeded a density of at 1 × 10^4^ cells per well for the cell viability assay. Finally, CCK‐8 was added and incubated for 2 h. A multimode reader was used to measure and record the absorbance at 450 nm.

### Clinical Body Test

The sensor was applied to the skin of volunteer to detect the signals from the nearside of brachial artery and ankle artery (Keithley 2000, OH USA and DIGILENT Analog Discovery 2, WA USA). The PW recorded via knitted strain sensor were compared with the signals from the same subject detected by a non‐invasive vascular screening device (ORMON BP‐203RPE III VP‐1000P, Japan).

The protocol for human clinical body test was approved by Ethical Committee of The Sixth People's Hospital of Nantong, Jiangsu, China (Affiliated Nantong Hospital of Shanghai University, Approval No. NTLYLL2023011). Prior to conducting pulse wave monitoring body tests to each individual, the physician read the informed consent form in a language that the recipient could understand, and the patient's consent was obtained. The duration of the experiment was less than 5 minutes for each individual. The single was acquired via the same method with mechanical and electrical evaluation test with 200 Hz sampling frequency (DIGILENT Analog Discovery 3, WA USA).

## Conflict of Interest

The authors declare no conflict of interest.

## Supporting information



Supporting Information

## Data Availability

The data that support the findings of this study are available from the corresponding author upon reasonable request.
