# Supplementary material for: Mechanical Compatibility in Stitch Configuration and Sensor Adhesion for High‐Fidelity Pulse Wave Monitoring
Source: Adv Sci (Weinh). 2025 Feb 14;12(14):2415608. doi: 10.1002/advs.202415608 (PMC11984903; doi:10.1002/advs.202415608)
Supplement: Supplementary file 1 — Supporting Information [file ADVS-12-2415608-s001.docx]

**Supporting Information**

**Mechanical Compatibility in Stitch Configuration and Sensor Adhesion for High-Fidelity Pulse Wave Monitoring**

*Zhongda Chen^#^, Jun Song^#^*, Yu Lu, Jing Zhu, Hongxu Zhu, Wenxian Du*, Benhui Hu**

Z. Chen, B. Hu

School of Biomedical Engineering and Informatics, Nanjing Medical University, 101 Longmian Avenue, Nanjing 211166, China

E-mail: [hubenhui@njmu.edu.cn](mailto:hubenhui@njmu.edu.cn) (Prof. B. Hu)

W. Du, H. Zhu

Institute of Diagnostic and Interventional Radiology, Shanghai Sixth People's Hospital, School of Medicine, Shanghai Jiaotong University, 600 Yishan Road, Xuhui, Shanghai, 200233, China

Email: [wx0910@mail.ustc.edu.cn](mailto:wx0910@mail.ustc.edu.cn) (Dr. W. Du)

J. Song

Materdicine Lab, School of Life Sciences, Shanghai University, 99 Shangda Road, Shanghai 200444, China

E-mail: [junsong@shu.edu.cn](mailto:junsong@shu.edu.cn) (Dr. J. Song)

J. Zhu

Department of Geriatrics, Affiliated Nantong Hospital of Shanghai University (The Sixth People's Hospital of Nantong), Shanghai University, 500 Yonghe Road, Nantong 226011, China

Y. Lu

School of Mechanical Engineering, Nantong University, 9 Seyuan Road, Nantong 226002, China

#These authors contributed equally to this work.

- Experimental Sections

1. Material characterization

Raman spectroscopy (Horiba LabRAM Evolution HR, Japan) and Fourier-transform infrared spectroscopy (FTIR, THERMO NICOLET 5700, USA) were applied to characterize the functional groups of the pristine fabric, the fabric with GO and the fabric with reduced GO (rGO).

The dynamic changes in water contact angle were tested using the Krüss Optronic DSA100 (Krüss Optronic, Germany) to characterize the surface property change during coating and reduction.

Attenuated total reflectance Fourier-transform infrared (ART-FTIR, THERMO NICOLET IS50, USA) was used to characterize the functional groups of the PAAm hydrogel.

Small-angle X-ray scattering (SAXS, Xenocs Xeuss3.0, France) and differential scanning calorimetry (DSC, TA Q2000, USA) were applied to characterize the crosslink degree of the PAAm hydrogel.

1. Sensor calibrations

To calibrate the sensor properties between the fabric strain and the relative resistance change, a series of tests was conducted (Instron 3344L3927, Germany), including: an initial calibration test that measured a one-way stretch up to a 60%/30% (plain/rib) strain; a reliability test to test in which data were recorded continuously during the stretching of the samples each for 10 cycles at 0-3%, 0-9% and 0-15%, and during stretch-release at frequencies of 0.125, 0.25, 0.5, 1 and 1.25 Hz; a strain-restore test for 0-5%, 0-10%, 0-15%, 5-10% and 10-15%; a durability test by testing 1,000 cycles at a 0-10% strain; a step response test involving a continuous stretch-restore and stop at the 3%, 6%, 9%, 12% and 15% point, with each stop point having five cycle repeats; a response time test to determine the maximum/minimum resistance acquired during a one-way 0.1% stretch using the fastest speed of the machine (500 HZ, stretch until a 0.5% strain over 2 ms).

All samples were tested in phosphate-buffered saline solution, deionized water and air, and the results were obtained using a tensile machine (stretch rate settings at 3%/s and samples size of 30 mm × 15mm, with a 5 mm × 15 mm area at the top and bottom dressed in silver paint and a digital multi-meter (Keithley 2000, USA).

1. *In vitro* sensor function evaluation

For the PW velocity (PWV) *in vitro* measurement, two identical sensors were mounted onto the dead-end tubing; the distance between these two sensors was 40 cm. The PW generator produced 10 signals with a 1 HZ frequency. The signal recorded by the two sensors was collected and measured by LabVIEW (National Instruments, USA). The time lag of the two starting points was recognized as the time required for the wave to transfer from one sensor to the other. Each test was applied to each dead-end tubing with different stiffness to simulate a healthy artery and a stiff artery.

1. Permeability test

The permeability of knitted fabric and the fabric/hydrogel dual layered structures were used for moisture permeability measurements. The tests were conducted according to the GB/T 12704.2-2009 standard. The samples were sealed in a moisture-permeable cup containing distilled water, placed in a sealed environment at 38±2 °C and relative humidity 50±2 %. After one hour of moisture transmission, the total weight of the moisture-permeable cup and sample was measured as m1. The temperature and humidity were maintained stable, and after an additional hour, the total weight was measured again as m2. The difference between m1 and m2, denoted as Δm, represents the moisture permeability. For the knitted fabric sample, the moisture permeability was Δm = 0.541 g; for the knitted fabric/hydrogel dual layer sample, the moisture permeability was Δm = 1.656 g. Therefore, the experimental results indicate that the fabric/hydrogel dual layer sample released more water vapor within the same time period, demonstrating the dynamic moisture and heat management ability of the fabric/hydrogel structure.

- Theoretical discussion

Based on dimensional analysis, we have developed some qualitative theoretical insights: For the mechanical interaction between the knitted fabric and the hydrogel layer, the following key physical quantities are identified:

The periodic strain $\boldsymbol{\gamma(t)}$ is characterized by two physical quantities: the maximum value $\boldsymbol{\gamma}_{\boldsymbol{x}}$ and the period $\boldsymbol{t}_{\boldsymbol{p}}$, which have the dimension of time $\boldsymbol{T}$.

Under cyclical loading, the total deformation energy of the entire dual-layer sensor consists of three parts:

$\boldsymbol{W}$=$\boldsymbol{W}_{\boldsymbol{f}}\mathbf{+}\boldsymbol{W}_{\boldsymbol{h}}\mathbf{+}\boldsymbol{W}_{\boldsymbol{h}\mathbf{,}\boldsymbol{\eta}}$

Expressed in terms of stiffness, we can express total deformation energy as:

$\boldsymbol{E}\mathbf{=}\boldsymbol{W}\mathbf{/}\boldsymbol{\gamma}_{\boldsymbol{x}}$=$\boldsymbol{E}_{\boldsymbol{f}}\mathbf{+}\boldsymbol{E}_{\boldsymbol{h}}\mathbf{+}\boldsymbol{E}_{\boldsymbol{h}\mathbf{,}\boldsymbol{\eta}}$

where the subscript f refers to the knitted fabric, and h refers to the hydrogel. The subscript $\boldsymbol{\eta}$ refers to the equivalent stiffness caused by the internal friction of the hydrogel.

Considering the viscosity of the hydrogel, the dimension of $\boldsymbol{\eta}$ is $\frac{\boldsymbol{M}}{\boldsymbol{TL}}$, where M is mass, T is time, and L is length. The dimension of the hydrogel stiffness $\boldsymbol{E}$ is $\frac{\boldsymbol{M}}{\boldsymbol{T}^{\mathbf{2}}\boldsymbol{L}}$, i.e., mass divided by the square of time and length.

Based on dimensional analysis, we can assume $\boldsymbol{E}_{\boldsymbol{h}\mathbf{,}\boldsymbol{\eta}}\boldsymbol{\propto}\frac{\boldsymbol{\eta}}{\boldsymbol{t}_{\boldsymbol{p}}}$, which means that stiffness is proportional to the viscosity and inversely proportional to the period. We assume that $\boldsymbol{E}_{\boldsymbol{\eta}}\mathbf{=}\boldsymbol{\alpha}\frac{\boldsymbol{\eta}}{\boldsymbol{t}_{\boldsymbol{p}}}$, where α is a dimensionless constant.

Assumption 1: Consideration of Hydrogel Alone

We assume that $\boldsymbol{E}_{\boldsymbol{f}}$ is independent of the crosslinking degree $\boldsymbol{\mu}$.

The equivalent stiffness is:

$\boldsymbol{E}_{\boldsymbol{equi}}\mathbf{=}\boldsymbol{E}_{\boldsymbol{f}}\mathbf{+}\boldsymbol{E}_{\boldsymbol{h}}\mathbf{+}\boldsymbol{E}_{\boldsymbol{\eta}}\mathbf{=}\boldsymbol{E}_{\boldsymbol{f}}\mathbf{+}\boldsymbol{E}_{\boldsymbol{h}}$+$\boldsymbol{\alpha}\frac{\boldsymbol{\eta}}{\boldsymbol{t}_{\boldsymbol{p}}}$

As the crosslinking degree $\boldsymbol{\mu}$ increases, $\boldsymbol{E}_{\boldsymbol{h}}$ increases while $\boldsymbol{\eta}$ decreases. This leads to a maximum value for $\boldsymbol{E}_{\boldsymbol{h}\mathbf{,}\boldsymbol{equi}}$.

Assumption 2: "De-adhesion" between Hydrogel and Fabric

Referring to the latest work in *Phys. Rev. Lett.* 2024, 133, 248201 by Samuel Poincloux *et al.*, the maximum deformation energy of the knitted structure, $\boldsymbol{W}_{\boldsymbol{f}}\mathbf{(}\boldsymbol{\gamma}_{\boldsymbol{x}}\mathbf{,}\boldsymbol{\gamma}_{\boldsymbol{y}}\mathbf{)}$, depends on strains in both the X and Y directions. In the relevant literature, the plot of $\boldsymbol{W}_{\boldsymbol{f}}\mathbf{(}\boldsymbol{\gamma}_{\boldsymbol{x}}\mathbf{,}\boldsymbol{\gamma}_{\boldsymbol{y}}\mathbf{)}$ exhibits an energy valley. In other words, $\boldsymbol{E}_{\boldsymbol{f}}$ will depend on $\boldsymbol{\gamma}_{\boldsymbol{y}}$.

When the hydrogel and fabric deform synchronously, the ratio of $\boldsymbol{\gamma}_{\boldsymbol{x}}$ to $\boldsymbol{\gamma}_{\boldsymbol{y}}$ is assumed to be the Poisson's ratio of the hydrogel, $\boldsymbol{\lambda}_{\boldsymbol{h}}$. In this case, the maximum deformation energy of the fabric depends only on $\boldsymbol{\gamma}_{\boldsymbol{x}}$, i.e., $\boldsymbol{W}_{\boldsymbol{f}}\mathbf{(}\boldsymbol{\gamma}_{\boldsymbol{x}}\mathbf{,}\boldsymbol{\lambda}_{\boldsymbol{h}}\boldsymbol{\gamma}_{\boldsymbol{x}}\mathbf{)}$.

Once the hydrogel and fabric begin to "de-adhere" with increasing crosslinking degree $\boldsymbol{\mu}$, $\boldsymbol{\gamma}_{\boldsymbol{y}}\mathbf{<}\boldsymbol{\lambda}_{\boldsymbol{h}}\boldsymbol{\gamma}_{\boldsymbol{x}}$, causeing $\boldsymbol{W}_{\boldsymbol{f}}$ to decrease. In other words, the equivalent stiffness of the fabric, $\boldsymbol{E}_{\boldsymbol{f}\mathbf{,}\boldsymbol{equi}}$, decreases. We assume a dimensionless parameter $\boldsymbol{\beta=}\frac{\boldsymbol{E}_{\boldsymbol{f}\mathbf{,}\boldsymbol{equi}}}{\boldsymbol{E}_{\boldsymbol{f}}}$, which is a decreasing function of the crosslinking degree $\boldsymbol{\mu}$.

For the total equivalent stiffness, we assume:

$\boldsymbol{E}_{\boldsymbol{equi}}\mathbf{=}\boldsymbol{E}_{\boldsymbol{h}}$+$\boldsymbol{\alpha}\frac{\boldsymbol{\eta}}{\boldsymbol{t}_{\boldsymbol{p}}}\mathbf{+}\boldsymbol{E}_{\boldsymbol{f}\mathbf{,}\boldsymbol{equi}}$=$\boldsymbol{E}$+$\boldsymbol{\alpha}\frac{\boldsymbol{\eta}}{\boldsymbol{t}_{\boldsymbol{p}}}\boldsymbol{+\beta}\boldsymbol{E}_{\boldsymbol{f}}$

where $\boldsymbol{\alpha}$, $\boldsymbol{t}_{\boldsymbol{p}}$, and $\boldsymbol{E}_{\boldsymbol{f}}$ are constant independent of the crosslinking degree $\boldsymbol{\mu}$, while $\boldsymbol{\eta}$ and $\boldsymbol{\beta}$ decrease with increasing $\boldsymbol{\mu}$, and $\boldsymbol{E}_{\boldsymbol{h}}$ increases. This leads to a maximum value for $\boldsymbol{E}_{\boldsymbol{equi}}$ as a function of $\boldsymbol{\mu}$.

- **Supporting Figures**


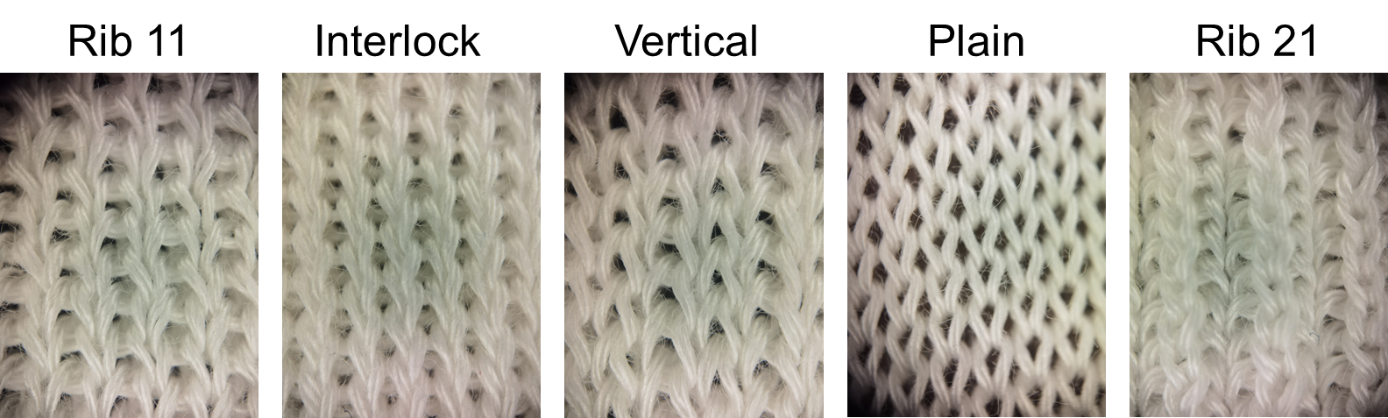


**Figure S1.** The optical images of five knitted sensors fabricated by 14-gauge machine.


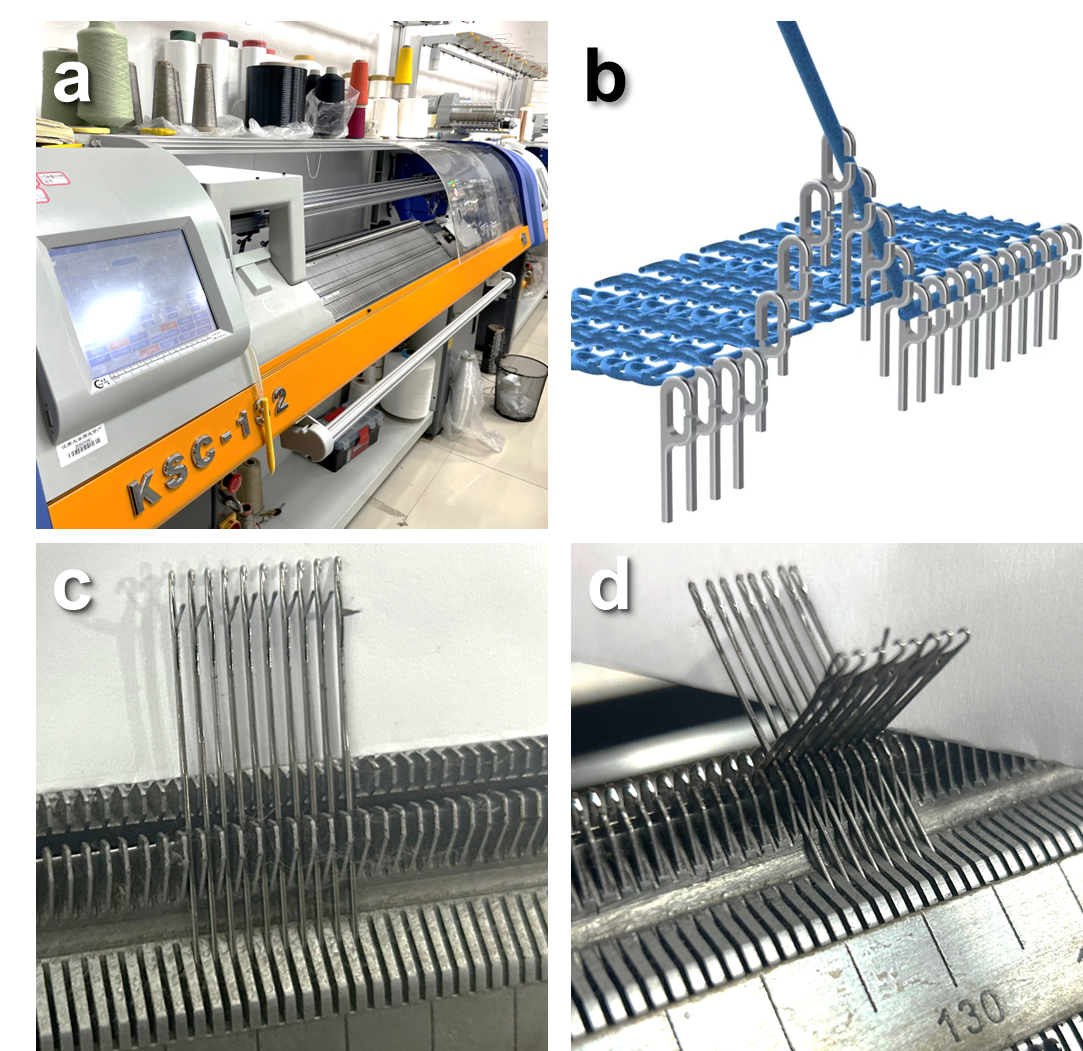


**Figure S2.** The knitting machine used for the knitted sensors fabrication.


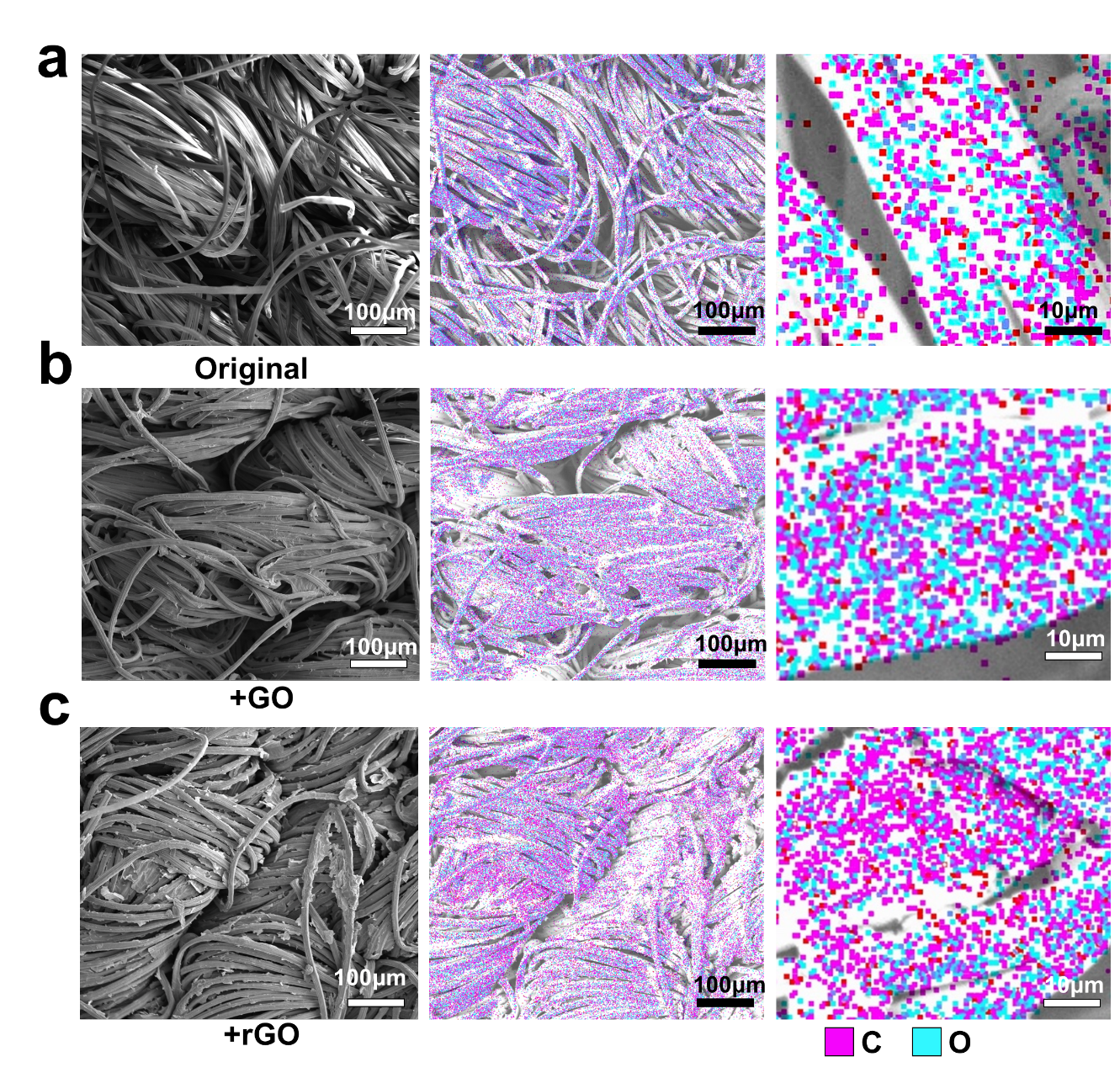


**Figure S3.** EDX mapping results for (a) pure fabric, (b) fabric coated with GO, (c) fabric coated with rGO.

**
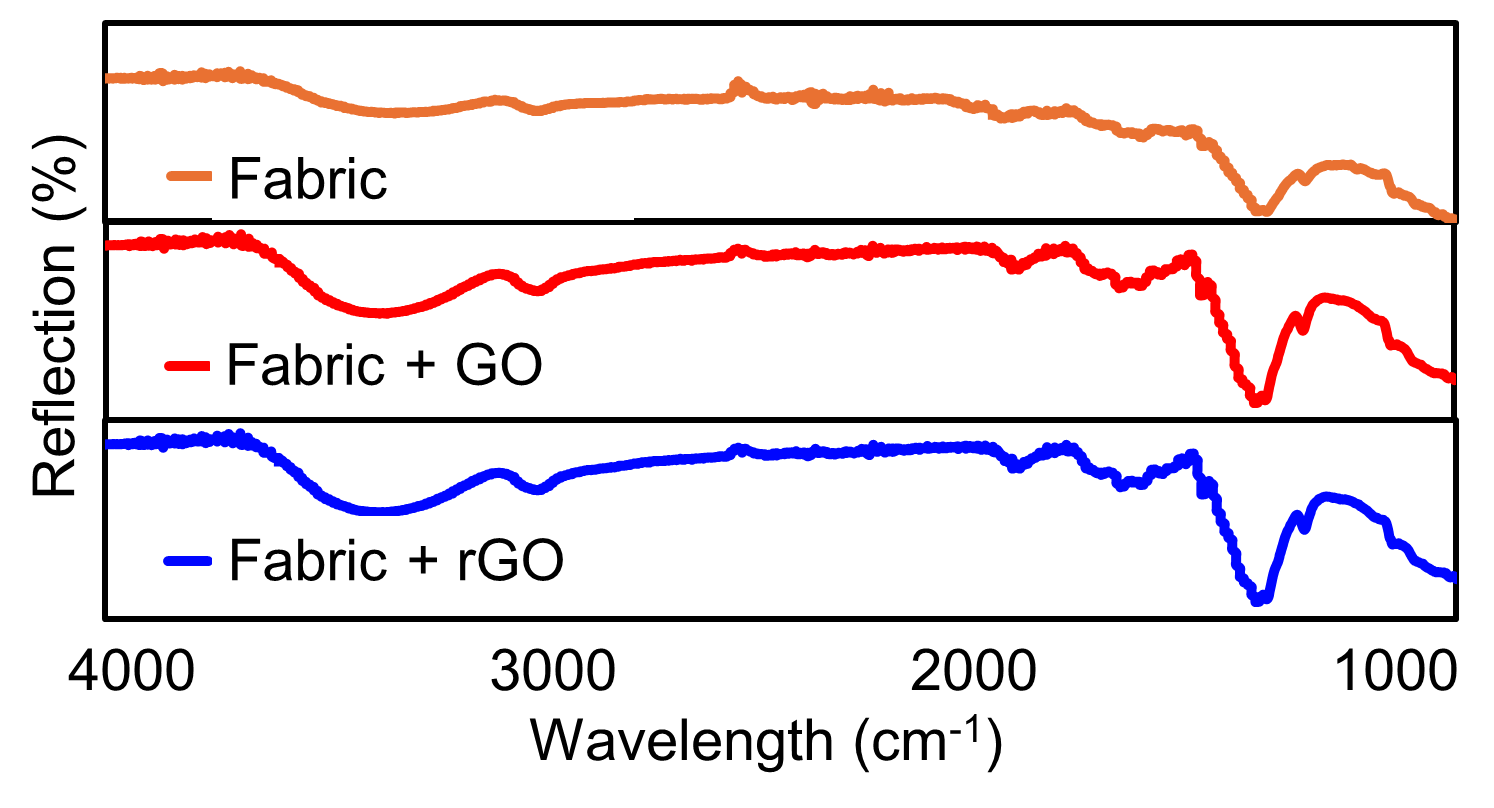
**

**Figure S4.** FT-IR results for pristine, GO-coated and rGO-coated knitted sensor.

**
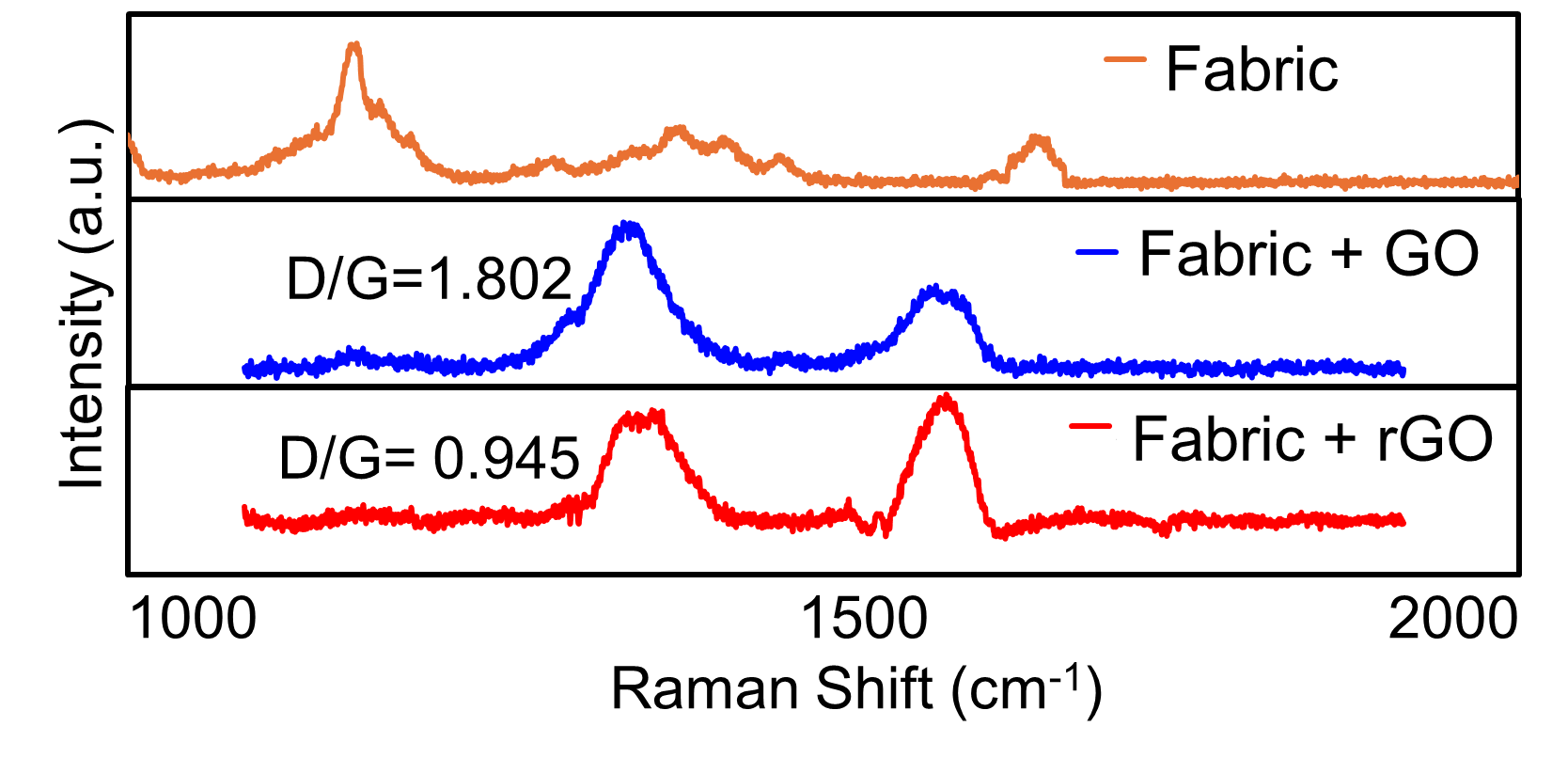
**

**Figure S5.** Raman results for pristine, GO-coated and rGO-coated knitted sensor.


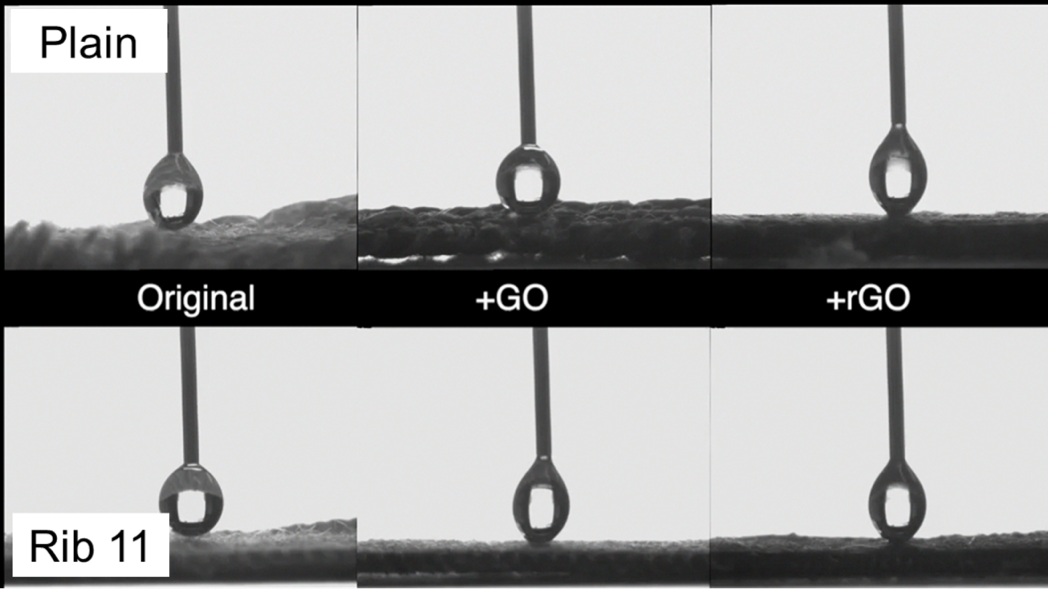


**Figure S6.** Dynamic water contract angle image.


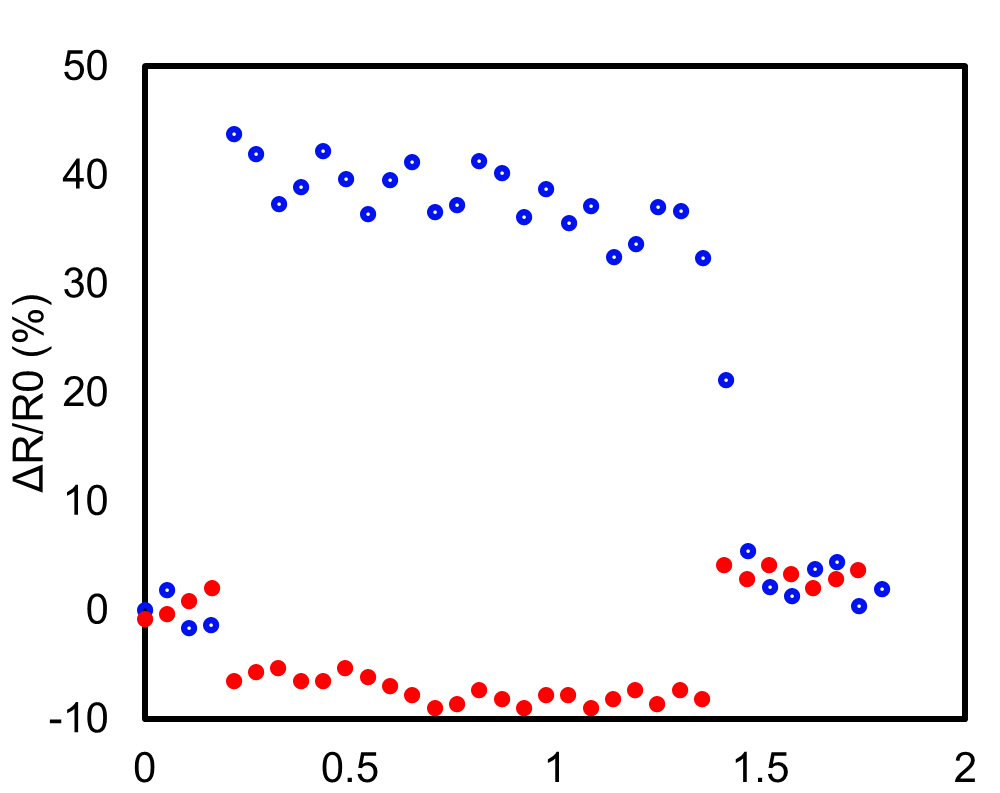


**Figure S7.** The real-time relative resistance changes of the sensor when subject to a fast-speed 5% strain test for electromechanical properties of the PWs sensor in the air.


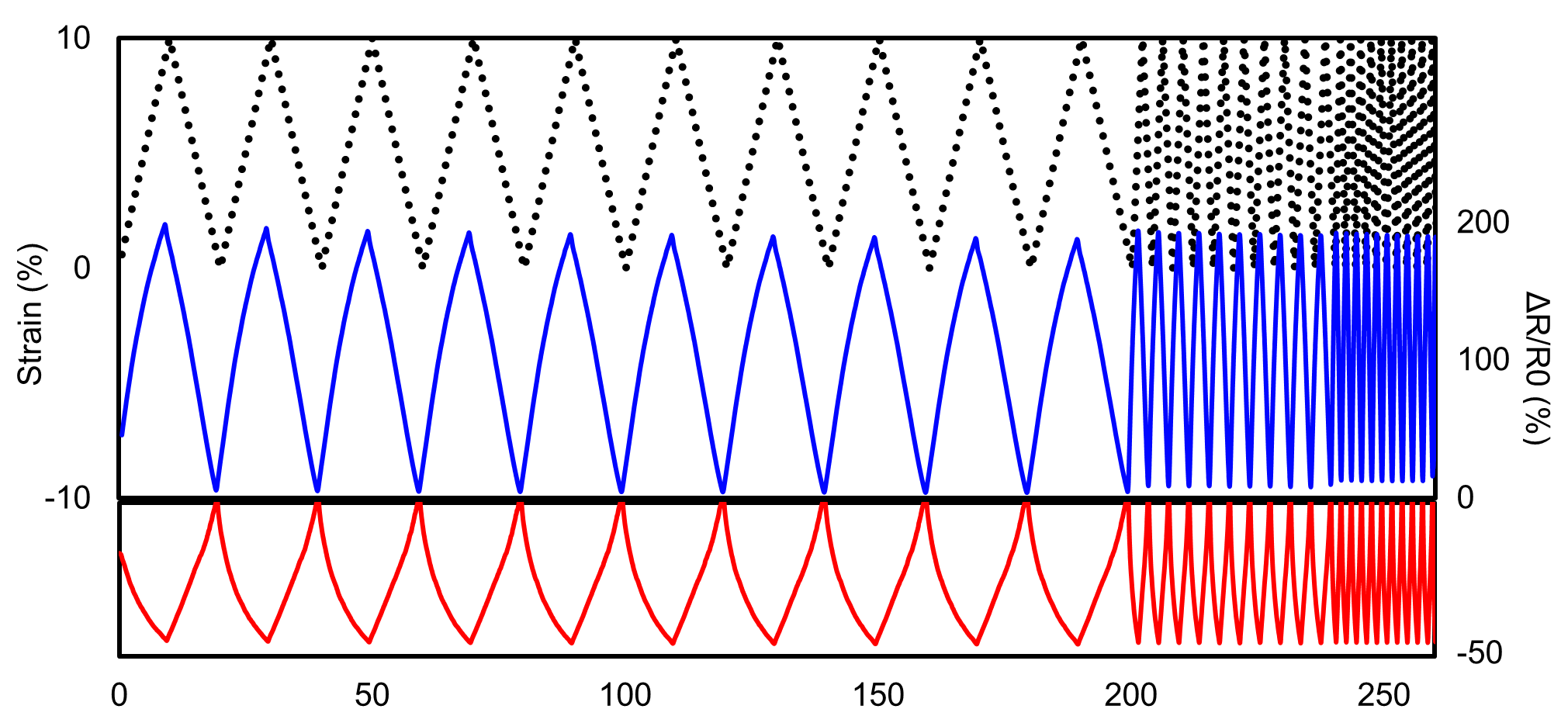


**Figure S8.** The reliability test for electromechanical properties of the PWs sensor in the air.


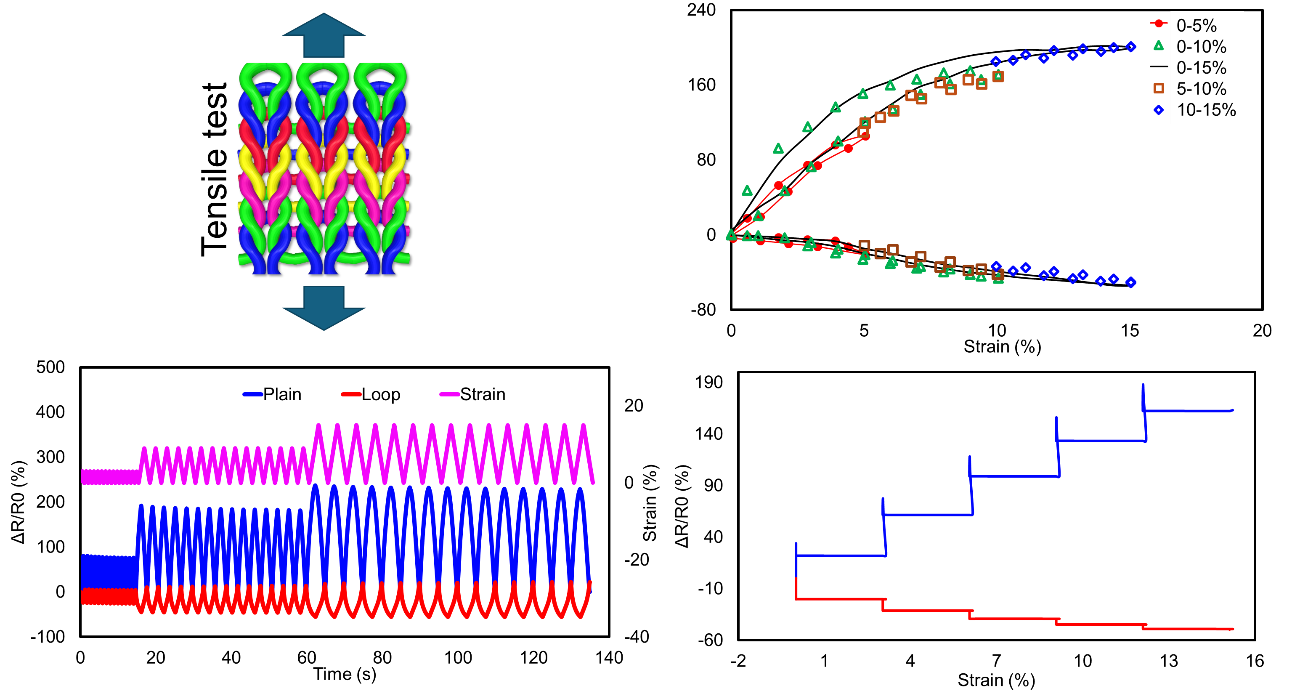


**Figure S9.** The calibration for the PWs sensor: the sensor is calibrated via tensile test.


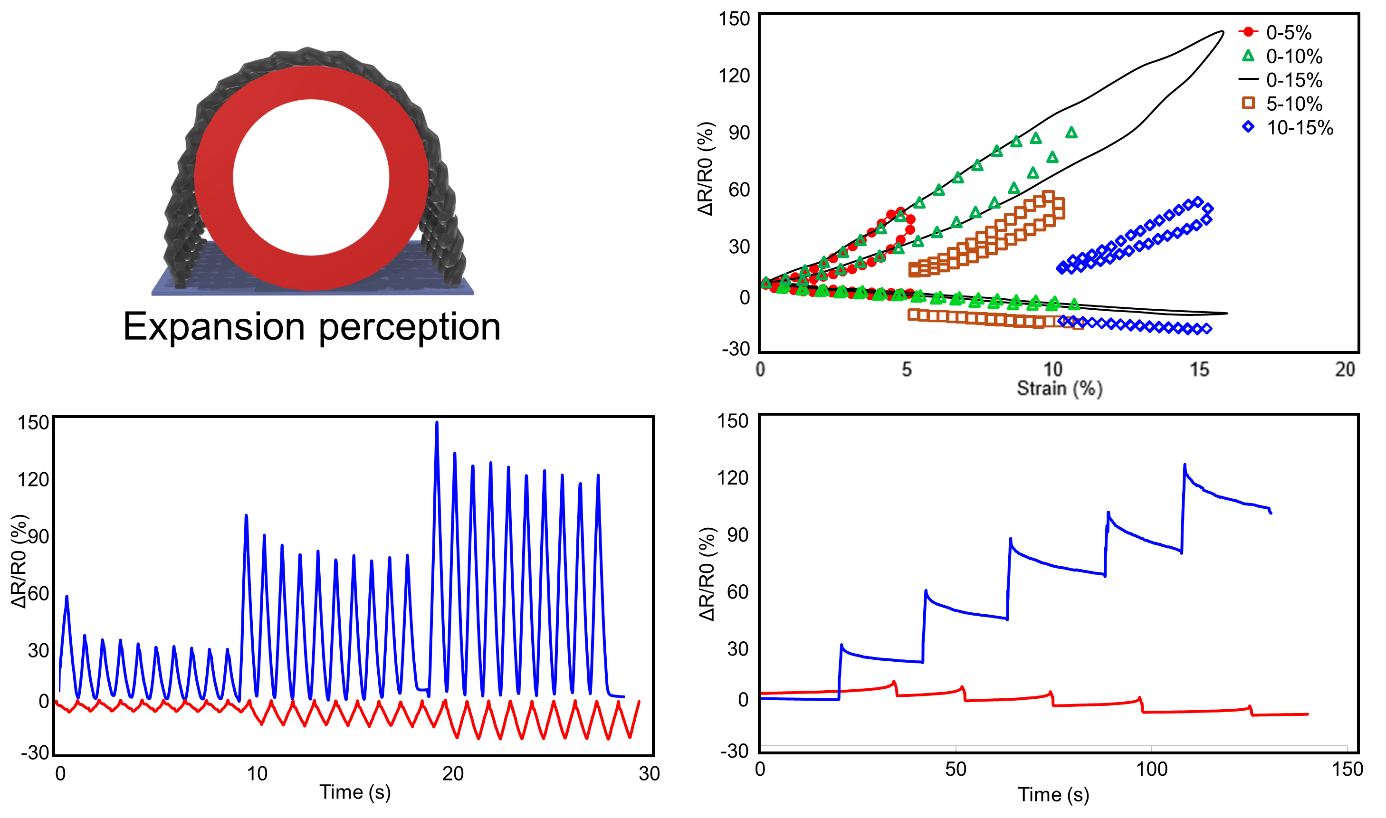


**Figure S10.** The calibration for the PWs sensor: the sensor is calibrated via tube expansion.


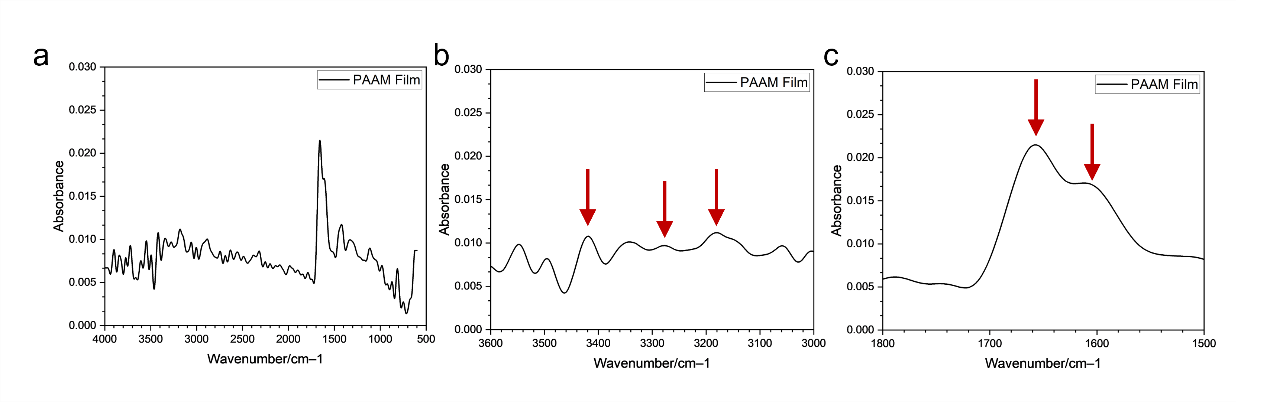


**Figure S11.** ART-FTIR spectra of PAAm hydrogels.

PAAm hydrogel films were prepsared by drying the hydrogel at 80 °C and obtained ART-FTIR spectra, as shown in Figure S11a. Figure S11b displays the ATR-FTIR spectrum of the hydrogel in the N–H stretching region. Three characteristic peaks centred at 3440, 3330, and 3200 cm^−1^ were observed. The bands at 3330 and 3200 cm^−1^ can be attributed to the asymmetric and symmetric stretching of the NH2 groups, respectively. Figure S11c shows the ATR-FTIR spectrum of hydrogel in the C=O stretching region. A strong C=O stretching band (amide I) at 1647 cm^−1^ and an N–H bending band (amide II) centred at 1605 cm^−1^ were observed.


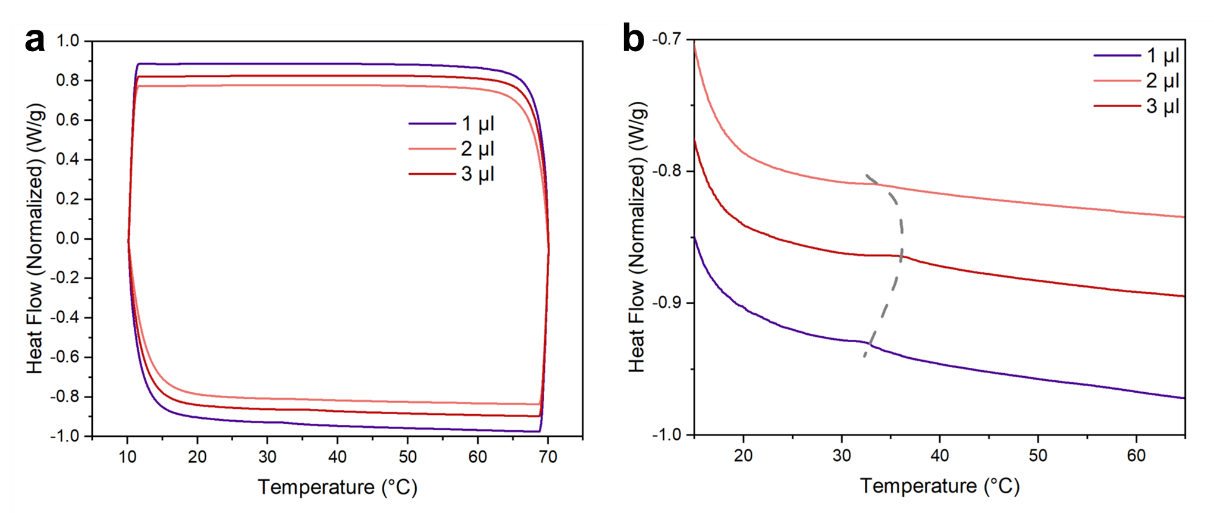


**Figure S12.** DSC curves of PAAm hydrogels with different TEMED volume.

The calorimetric measurements conducted in our study reveal that as the concentration of TEMED increases, the phase transition temperature of the PAAm hydrogel rises, which indicates an increase in the phase transition enthalpy. We therefore infer that the addition of more TEMED enhances the crosslinking density within the hydrogel.


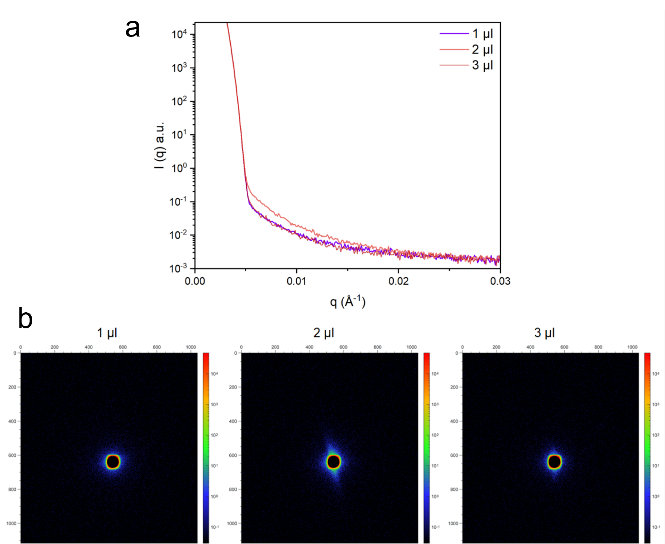


**Figure S13.** SAXS spectra and 2D patten of PAAm hydrogel with different TEMED volume.

This result reveals that PAAm gels crosslinked with different TEMED concentrations show scattering at low q regions, with only the 2 µL group exhibiting more significant scattering. The intensity in the low q region for this group increased by approximately half an order of magnitude. This can be attributed to phase separation induced by hydrogen bonding interactions, leading to the formation of large aggregates. Therefore, the 2 µL sample demonstrates improved mechanical properties, which is consistent with the conclusion drawn in our manuscript.


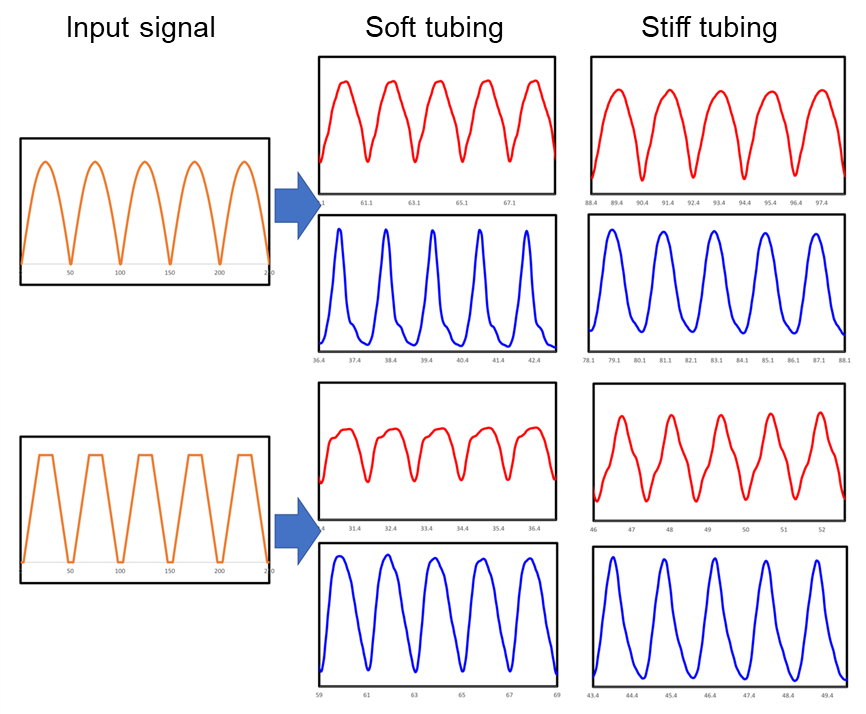


**Figure S14.** Comparison of the sine wave and the trapezoidal wave between tubing with different stiffness from two knitting strain sensors.


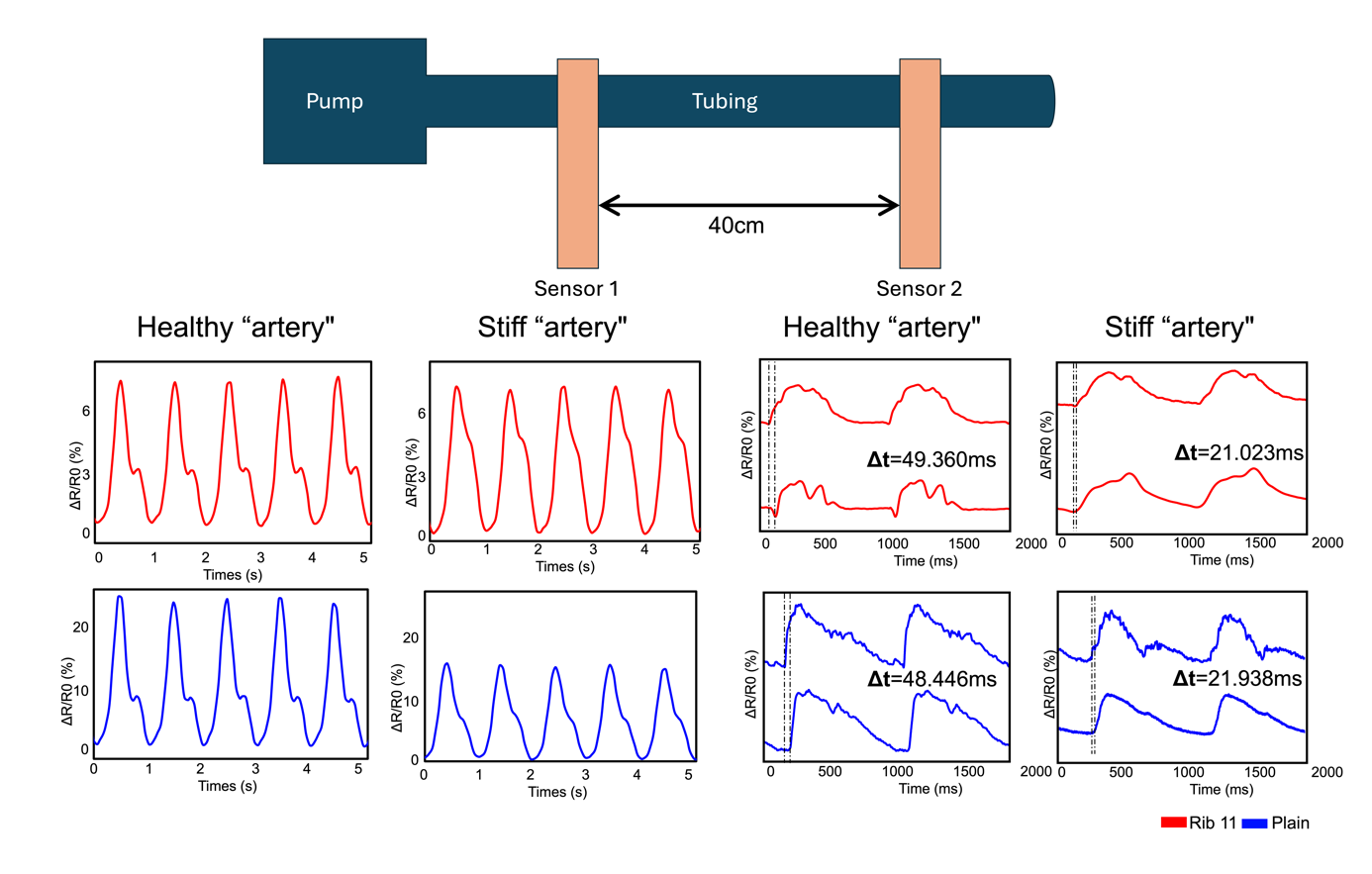


**Figure S15.** The knitting sensor being able to detect pulse wave velocity (PWV) via calculating the time delta of starting point between two waveforms recorded. Both of Rib11 and Plain sensors recorded a similar time lag for the “healthy artery” and the “stiff artery”. By using this method, it is possible to conveniently determine the degree of artery stiffness by using our sensor. Compared with the recording of the PWs waveform at one signal point, the PWV can detect and diagnose artery stiffness at a macroscopic level.


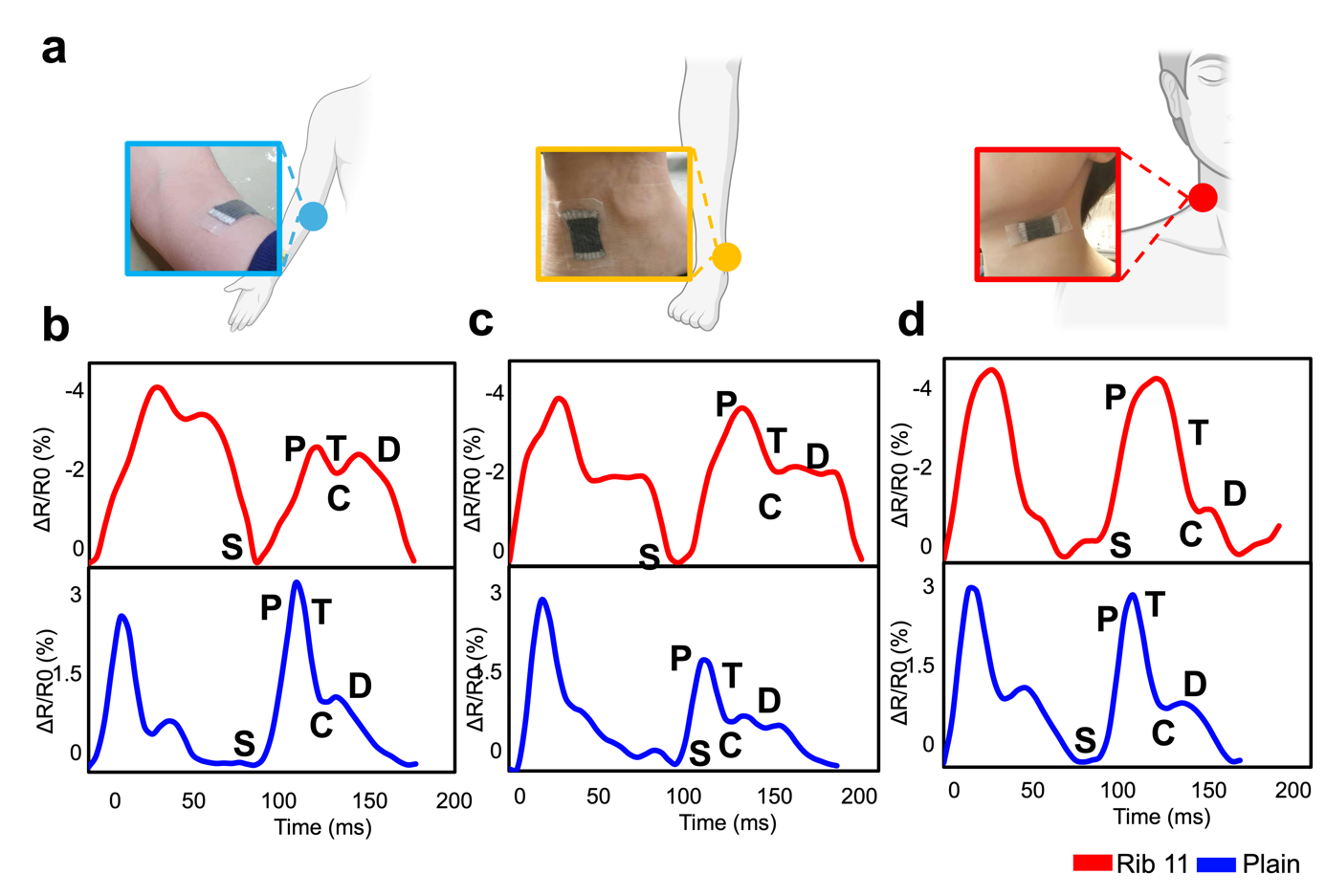


**Figure S16.** The PWs waveform record via knitting strain sensors (Rib11 and Plain) at neck, wrist and ankle.

- **Tables**

**Table S1. All patients’ information**

| **Patient No.** | | **Gender** | | **Age** | **Diagnoses** |
| --- | --- | --- | --- | --- | --- |
| 1 | Male | | 80 | | 1. Cerebral infarction,  2. Hyperlipidemia,  3. Liver insufficiency,  4. Chronic gastritis,  5. Reflux esophagitis,  6. Coronary atherosclerotic heart disease,  7. Pulmonary emphysema |
| 2 | Female | | 77 | | 1. Lacunar cerebral infarction,  2. Coronary atherosclerotic heart disease,  3. Hypertension grade 3 (very high risk),  4. Carotid plaque,  5. Chronic gastritis,  6. Dyspepsia,  7. Hypothyroidism,  8. Osteoporosis,  9. Insomnia,  10. Constipation |
| 3 | Female | | 72 | | 1. Chronic gastritis,  2. Hypertension grade 1 (high risk),  3. Lacunar cerebral infarction,  4. Hyperlipidemia |
| 4 | Female | | 56 | | 1. Lung infection,  2. Transient ischemic attack,  3. Hypokalemia,  4. Hyperlipidemia,  5. Chronic atrophic gastritis,  6. Vitamin D deficiency,  7. Osteoporosis |
| 5 | Female | | 88 | | 1. Lung infection,  2. Chronic bronchitis,  3. Lacunar cerebral infarction,  4. Coronary atherosclerotic heart disease,  5. First-degree atrioventricular block,  6. Heart failure,  7. Heart function grade III,  8. Hypertension grade 3 (very high risk),  9. Type 2 diabetes,  10. Venous insufficiency in lower limbs,  11. Mild anemia, 12. Hemorrhoids |
| 6 | Male | | 90 | | 1. Lung infection,  2. Coronary atherosclerotic heart disease,  3. Tricuspid valve regurgitation,  4. Heart function grade III,  5. Lacunar cerebral infarction,  6. Hypertension grade 3 (very high risk),  7. Type 2 diabetes,  8. Hypertriglyceridemia,  9. Hypoproteinemia,  10. Reflux esophagitis, 11. Carotid plaque,  12. Deep vein thrombosis in lower limbs,  13. Prostatic hyperplasia,  14. Urinary tract infection |
| 7 | Female | | 86 | | 1. Cerebral infarction,  2. Coronary atherosclerotic heart disease,  3. Sinus bradycardia,  4. Hypertension grade 3 (very high risk),  5. Osteoporosis,  6. Hyperlipidemia,  7. Fatty liver |
| 8 | Female | | 82 | | 1. Lacunar cerebral infarction,  2. Chronic gastritis,  3. Reflux esophagitis,  4. Lung infection,  5. Pulmonary emphysema,  6. Renal insufficiency,  7. Hypertension grade 3 (very high risk),  8. Hyperlipidemia,  9. Hyperuricemia,  10. Hypocalcemia,  11. Hypoproteinemia,  12. Mild anemia,  13. Carotid plaque,  14. Thyroid nodule |
| 9 | Female | | 83 | | 1. Erysipelas,  2. Coronary atherosclerotic heart disease,  3. Heart failure,  4. Heart function grade III,  5. Lacunar cerebral infarction,  6. Hypertension grade 3 (very high risk),  7. Chronic obstructive pulmonary disease with acute exacerbation,  8. Lung infection,  9. Hypoxemia,  10. Folic acid deficiency,  11. Kidney stone |
| 10 | Female | | 89 | | 1. Coronary atherosclerotic heart disease,  2. Heart failure,  3. Heart function grade III,  4. Premature ventricular contraction,  5. Lung infection, 6. Hypoxemia,  7. Lacunar cerebral infarction,  8. Fatty liver, 9. Hyperlipidemia,  10. Osteoporosis,  11. Vitamin D deficiency,  12. Urinary tract infection,  13. Vitamin B12 deficiency |
| 11 | Female | | 83 | | 1. Venous insufficiency in lower limbs,  2. Stasis dermatitis (right lower limb),  3. Coronary atherosclerotic heart disease,  4. Heart failure,  5. Heart function grade III,  6. Moderate mitral valve regurgitation,  7. Lacunar cerebral infarction,  8. Thrombocytosis,  9. Hypertension grade 2 (very high risk),  10. Type 2 diabetes,  11. Hyperlipidemia,  12. Hypoxemia,  13. Panniculitis,  14. Post-cholecystectomy state,  15. Urinary tract infection |
| 12 | Female | | 68 | | 1. Osteoporosis,  2. Coronary atherosclerotic heart disease,  3. Chronic bronchitis,  4. Hypertension grade 3 (very high risk),  5. Hypertriglyceridemia,  6. (Left) simple renal cyst,  7. Chronic gastritis,  8. Reflux esophagitis,  9. Fatty liver,  10. Thyroid nodule,  11. Insomnia |
| 13 | Female | | 72 | | 1. Coronary atherosclerotic heart disease,  2. Lacunar cerebral infarction,  3. Chronic gastritis,  4. Reflux esophagitis,  5. Constipation,  6. Insomnia,  7. Osteoporosis |
| 14 | Male | | 79 | | 1. Cerebral infarction,  2. Coronary atherosclerotic heart disease,  3. Hypertension grade 3 (very high risk),  4. Fatty liver,  5. Choledocholithiasis,  6. Prostatic hyperplasia |
| 15 | Male | | 69 | | 1. Lacunar cerebral infarction,  2. Coronary atherosclerotic heart disease,  3. Lung infection,  4. Type 2 diabetes,  5. Carotid plaque,  6. Left kidney stone,  7. Prostatic hyperplasia,  8. Appendectomy,  9. Cervical disc herniation |
| 16 | Female | | 68 | | 1. Osteoporosis,  2. Coronary atherosclerotic heart disease,  3. Lacunar cerebral infarction,  4. Hypertension grade 3 (very high risk),  5. Liver insufficiency,  6. Hyperlipidemia,  7. Mild anemia,  8. Anxiety and depression,  9. Constipation,  10. Hypothyroidism,  11. Chronic gastritis,  12. Reflux esophagitis,  13. Urinary tract infection |
| 17 | Female | | 70 | | 1. Lacunar cerebral infarction,  2. Acute gastroenteritis,  3. Hypoproteinemia,  4. Hypokalemia |
